# Light modulates important physiological features of *Ralstonia pseudosolanacearum* during the colonization of tomato plants

**DOI:** 10.1038/s41598-021-93871-9

**Published:** 2021-07-15

**Authors:** Josefina Tano, María Belén Ripa, María Laura Tondo, Analía Carrau, Silvana Petrocelli, María Victoria Rodriguez, Virginia Ferreira, María Inés Siri, Laura Piskulic, Elena Graciela Orellano

**Affiliations:** 1 Instituto de Biología Molecular y Celular de Rosario, Facultad de Ciencias Bioquímicas y Farmacéuticas (IBR-FBIOyF), Consejo Nacional de Investigaciones Científicas y Técnicas, Universidad Nacional de Rosario (CONICET-UNR), Suipacha 531, S2002LRK Rosario, Argentina; 2 Facultad de Ciencias Bioquímicas y Farmacéuticas, Universidad Nacional de Rosario, Rosario, Argentina; 3 Área Biología Vegetal, Facultad de Ciencias Bioquímicas y Farmacéuticas, Universidad Nacional de Rosario, Rosario, Argentina; 4 Área Microbiología, Departamento de Biociencias, Facultad de Química, Universidad de la República, Montevideo, Uruguay; 5 Área Estadística y Procesamiento de datos, Facultad de Ciencias Bioquímicas y Farmacéuticas, Universidad Nacional de Rosario, Rosario, Argentina

**Keywords:** Microbiology, Plant sciences

## Abstract

*Ralstonia pseudosolanacearum* GMI1000 (*Rpso* GMI1000) is a soil-borne vascular phytopathogen that infects host plants through the root system causing wilting disease in a wide range of agro-economic interest crops, producing economical losses. Several features contribute to the full bacterial virulence. In this work we study the participation of light, an important environmental factor, in the regulation of the physiological attributes and infectivity of *Rpso* GMI1000. In silico analysis of the *Rpso* genome revealed the presence of a *Rsp0254* gene, which encodes a putative blue light LOV-type photoreceptor. We constructed a mutant strain of *Rpso* lacking the LOV protein and found that the loss of this protein and light, influenced characteristics involved in the pathogenicity process such as motility, adhesion and the biofilms development, which allows the successful host plant colonization, rendering bacterial wilt. This protein could be involved in the adaptive responses to environmental changes. We demonstrated that light sensing and the LOV protein, would be used as a location signal in the host plant, to regulate the expression of several virulence factors, in a time and tissue dependent way. Consequently, bacteria could use an external signal and *Rpsolov* gene to know their location within plant tissue during the colonization process.

## Introduction

Light is an important environmental factor in all ecosystems because it is a source of energy and information. Almost all organisms can use light to sense their surroundings and thus be able to adapt to environmental changes, allowing them survival^1^.

Plant physiology is deeply regulated by environmental factors, being light probably one of the most relevant. As well as direct effects on plant metabolism, growth and development, light inevitably influences many other plant responses, including those induced by pathogen attack^2^. The role of light in host defense responses has been widely studied and it is known that an appropriate light environment is required for a full defense response^3–6^.

In phytopathogenic bacteria, light can define the result of plant-pathogen interactions, not only by affecting the plant's defense responses but also by modulating the virulence of the pathogens^7^. Recent reports revealed the light influence on bacterial lifestyle transitions, motility, and virulence^8^. Bacterial plant pathogens evolved to detect light conditions associated with different levels of plant resistance. *Xanthomonas citri* subsp. *citri* (*Xcc*) is a non-vascular hemibiotrophic phytopathogen responsible for citrus canker disease. *Xcc* physiology and its ability to colonize the host plant tissue are modulated by light perception^9^. In addition, *Pseudomonas syringae* pv. *tomato* DC3000 (*Psto*), another hemibiotrophic bacterium that causes bacterial speck in tomatoes, regulates its motility and virulence under different light conditions^10,11^. These bacteria, before the colonization of the host plant apoplast, grow epiphytically on the leaves surface having an important dose of solar radiation.

Light signals, their wavelengths, fluctuations in intensity and degree of polarization are perceived and transmitted by photoreceptor proteins. These proteins are classified into six different families: rhodopsins, phytochromes, photoactive proteins yellow (PYP, also called xanthopsins), LOV proteins (Light, Oxygen or Voltage), cryptochromes and BLUF (Blue-Light Sensing Using Flavin) proteins^7,12^. LOV proteins are a type of blue light photoreceptors, which are flavin binding proteins that use a flavin mononucleotide (FMN) as a chromophore^13^. The prominent role of LOV photoreceptor in the virulence processes of different pathogenic bacteria such as *Brucella abortus*^14,15^, *Pseudomonas syringae* pv. *syringae*^16^, *Pseudomonas syringae* pv. *tomato*^10,11,17^ and beneficial bacteria such as *Rhizobium leguminosarum*^18^, and *Mesorhizobium loti*^19^ was studied.

*Ralstonia solanacearum* (*Rso*) is a Gram negative β-proteobacteria responsible for multiple diseases related to the wilting of more than 200 plant species, causing huge economic losses worldwide, especially in developing tropical countries. This phytopathogen invades the vascular tissue in a systemic way^20,21^. Due to its wide range of hosts, large geographic distribution and diverse pathogenic behavior, this heterogeneous group is recognized today as a "species complex" (RSSC, *Ralstonia solanacearum* Species Complex)^22^. Within the RSSC, four subdivisions called phylotypes are recognized and each phylotype is divided into secuevars. Among the strains representing the phylotype I *R. pseudosolanacearum* GMI1000 (*Rpso* GMI1000) is found, a strain whose genome was completely sequenced^23^. Although *R. solanacearum* is considered a plant pathogen, it mainly behaves as a soil bacterium of saprophytic life with an extremely versatile lifestyle, which allows the bacteria to survive in the soil for long periods in the absence of its host plant. *Rso* moves toward the plant roots by different motilities such as swimming and twitching, searching for favorable growth conditions. After invading the host plant root system, the bacterium adheres to host cells and develops a biofilm to colonize the root cortex^24^. Then it reaches the vascular tissue spreading systemically to all plant tissues through the xylem. Finally, the exopolysaccharide (EPS) overproduction and bacterial active proliferation produce the obstruction of the xylem vessels, rendering the characteristic bacterial wilting phenotype, due to the lack of water and nutrients^25^.

In silico analysis of the *R. pseudosolanacearum* (*Rpso*) GMI1000 genome revealed the presence of a gene *Rsp0254* encoding a putative LOV protein, the only photoreceptor protein detected, which led us to hypothesize that light could influence *Rpso* lifestyle and its interaction with the host plant^23^.

A complex regulatory network that responds to environmental conditions controls the expression of virulence factors in *Rso*. The global regulator PhcA presents the largest regulon described to date in the *Rso* species complex that directly or indirectly controls the expression of many genes^26^. Furthermore, the type III secretion system (T3SS), encoded by the *hrp* cluster, that allows effector proteins translocation into plant cells, is a key determinant of pathogenicity required for the disease development in host plants. The HrpG transcription factor controls the expression of many genes that promote the bacterial adaptation to the plant, including detoxifying enzymes, phytohormones, lectins, metabolic enzymes and transporters^25^. In addition, HrpG also functions as an activator of *hrpB,* which induces the expression of the structural units of T3SS and its associated effectors^27,28^. *Rso* is also capable of perceiving signals derived from the host cell wall during initial bacterial-plant cell contact, activating the expression of *hrp* genes^29^. The VsrAD two-component system controls the transcription of genes involved in EPS synthesis and other traits, some of which contribute strongly to *Rso* ability to colonize tomato stems and multiply *in planta*, regardless of the effect of the regulator on the EPS production^30^. EPS is required in the early and the late disease stages, during root colonization and later xylem physical obstruction, since it forms the necessary structural scaffold required for biofilm formation in both stages. Biochemical and genetic studies indicate that EPS and the enzymes that degrade the plant cell wall are necessary for the complete virulence. *pehR* gene controls early virulence factors and is also a positive regulator of the swimming motility cascade^31^. In this context, the physiological base of the bacterial wilt disease is multifactorial. Besides HrpG and PrhG transcriptional regulators, the *Rso* regulation network also includes numerous well-studied regulators such as PhcA, PrhN, PrhO, and XpsR cascades^26^.

In this work, the involvement of light and LOV protein in the regulation of *Rpso* physiological attributes and infectivity was elucidated. With this aim, we constructed a mutant strain lacking a functional *Rpsolov* gene (*Rpso*Δ*lov*) and studied the effect of the absence of this gene on bacterial physiological characteristics. In addition, it was studied how certain environmental factors, such as light, affect the interaction between *Rpso* GMI1000 and its host plants. We demonstrated that light and the LOV protein control motility, adhesion and biofilm formation in *Rpso* allowing the successful colonization of the tomato plant rendering the bacterial wilt disease. This is the first report revealing the role of light of the vascular phytopathogen *Rpso* GMI1000.

## Results

### *Rsp0254* is a LOV type photoreceptor putative fused to diguanylate cyclase-phosphodiesterase (DGC-PDE) response regulator

The 5.8 Mbp genome of the model strain *R. pseudosolanacearum* GMI1000 (*Rpso* GMI1000) is fully sequenced and organized into two circular replicons: a 3.7 Mbp chromosome and a 2.1 Mbp megaplasmid^23^. According to the *in-silico* analysis of the *Rpso* genome, in the megaplasmid there is an open reading frame coding for a putative LOV domain protein *Rsp0254* (named *Rpsolov* gene for clarity purposes), a transmembrane predictive protein of 1178 amino acids. The *Rpsolov* gene presents different domains: a HAMP transmembrane signaling domain, a family of PAS domains that contain the LOV domain (635–738aa) (Supplementary Material 1 (S1)), and the domain responsible for regulating the response made up of a diguanylate cyclase (GGDEF) fused to a phosphodiesterase (EAL) domain^32,33^. In transmembrane proteins, the HAMP domains are found on the cytoplasmic side, where they convert intracellular transmembrane signals to response signals^34^. In the case of PAS domains, they can act as direct receptors or, as in the case of LOV domains, possess a cofactor responsible for the perception of light. LOV domains contain a molecule of flavin mononucleotide (FMN) as a non-covalently bound chromophore. The *Rpso* LOV protein presents a conserved key functional amino acid residue, the cysteine Cys 672, known to be important for photochemistry and signaling.

### The *Rpsolov* gene distribution in the *Ralstonia solanacearum* species complex (RSSC)

Multiple alignments of the deduced amino acid sequences of LOV proteins from representative strains belonging to the four phylotypes including: *Rpso* GMI1000 (phylotype I)^23^, *Rpso* strain OE1-1 (phylotype I)^35^, *Rpso* FQY_4 (phylotype I)^36^, *Rso* K60 (phylotype IIA)^37^, *Rso* CFBP2957 (phylotype IIA)^38^, *Rso* IPO1609 (phylotype IIB)^38^, *Rso* UW551 (phylotype IIB)^38^, *Rso* Po82 (phylotype IIB)^39^, *Rso* UY331 (phylotype IIB)^40^, *Rpso* CMR15 (phylotype III)^38^, *R. syzygii* R24 (phylotype IV)^41^ and *R. syzygii* PSI07 (phylotype IV)^38^ revealed that the LOV protein is present in all *Rso* strains sequenced and possess highly conserved domains suggesting that light would play an important role in the *Rso* free lifestyle and during the plant-interaction (Supplementary material S1).

### Different light conditions and *Rpsolov* gene deletion do not affect the growth of *Rpso*

**Figure 1:**
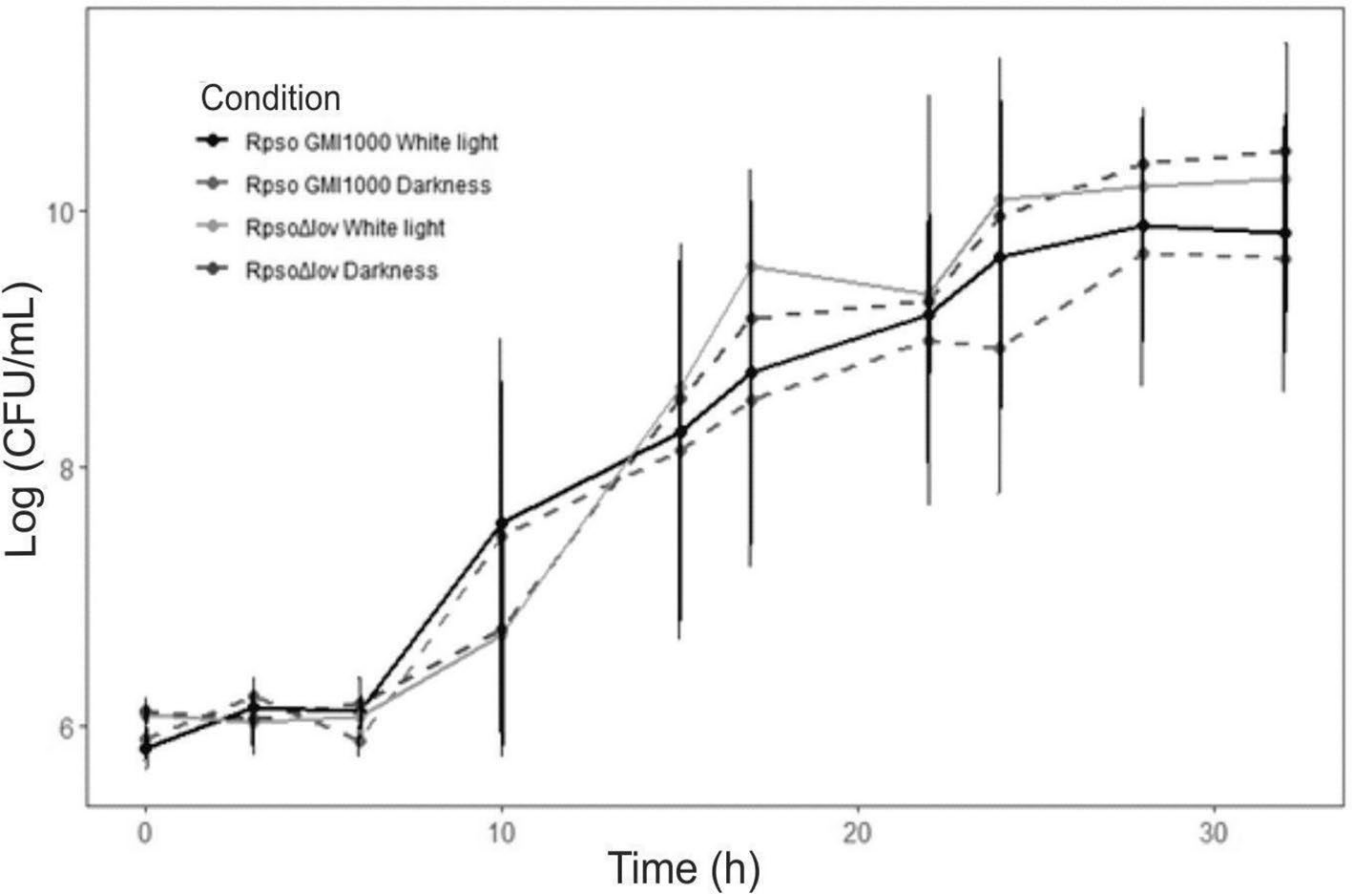
Growth curves of *Rpso* GMI1000 and *Rpso*Δ*lov* under different light conditions. Bacterial cells were cultured in BG medium at 28 °C under exposure to constant white light or darkness. Aliquots were taken at the indicated times and measured for colony-forming capacity by serial dilution and plating on BG-agar. Colonies were counted after 48 h incubation at 28 °C. No significant effect in the growth of *Rpso* GMI1000 or *Rpso*Δ*lov* was found under the two lighting conditions (p = 0.5727).

To determine whether light or the absence of the *Rpsolov* gene affect *Rpso* viability and growth kinetics, we analyzed the bacterial growth in white light and in darkness of *Rpso* GMI1000 and *Rps*oΔ*lov* strains. The CFU/mL of culture were determined at different periods, but no significant change in the growth of *Rpso* GMI1000 or *Rps*oΔ*lov* was found under the two lighting conditions tested (p = 0.5727) (Fig. 1).

### *Rpso* swimming and twitching motilities are regulated by light and by *Rpsolov* gene

**Figure 2:**
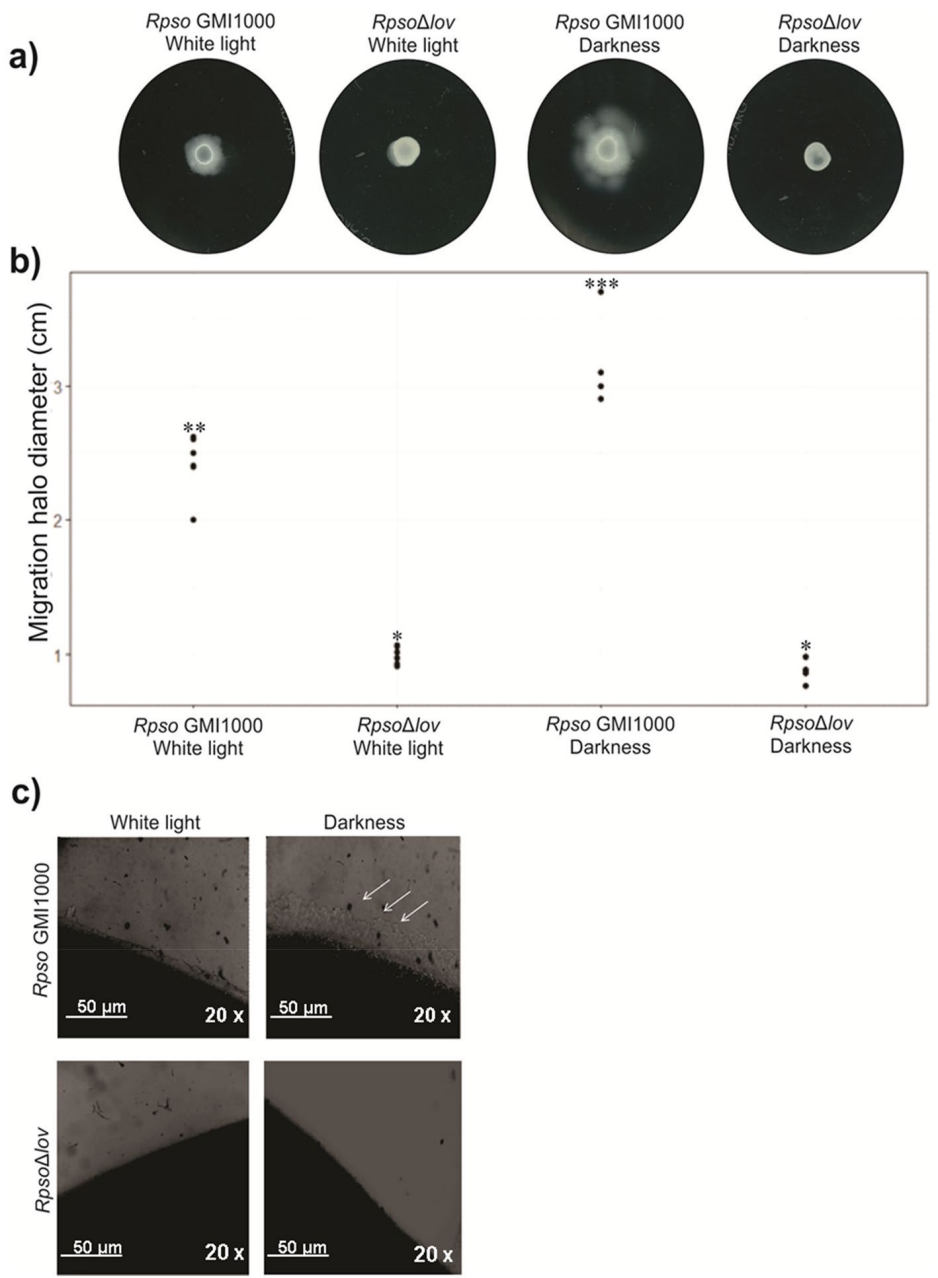
Effect of the *Rpsolov* gene mutation in swimming and twitching motility assays under different lighting conditions. (**a**) Representative images of both bacterial strains swimming agar plates incubated 48 h under white light or continuous darkness. (**b**) Measurement of the diameter of the bacterial migration halos (cm) from six independent experiments. Significant differences between conditions are represented by different asterisks (**-*p < 0.0001, ***-*p = 0.0002 and **-***p = 0.0004). (**c**) Images of *Rspo* GMI1000 and *Rpso*Δ*lov* colony edges observed through an optical microscope at a 20x magnification. White arrows indicate the borders of the colonies, observing the typical raft of this motility. Images are representative of four independent experiments.

*Rpso* is a motile bacterium with one to four polar flagella able to slide on liquid medium by swimming. This motility contributes to the full virulence of this bacterium in the early stages of host invasion and colonization^42^. To determine the effect of light on *Rpso* wild type and *Rps*oΔ*lov* swimming motility, bacteria were grown in white light and darkness (Fig. 2a), and the motility was quantified by measuring the diameter of the migration halo (Fig. 2b). *Rpso* GMI1000 produced smaller migration zones under white light compared to darkness (p = 0.0004). On the other hand, the mutant strain did not present swimming motility halos in the conditions assayed (p < 0.0001).

Twitching motility is a type IV pili-mediated translocation that allows bacterial adhesion to the plant roots^43^. The effect of light on *Rpso* twitching motility was evaluated in both bacterial strains under different lighting conditions. When the plates were incubated under darkness, colonies with layered edges and multiple irregular projections were observed which is typical of this type of bacterial motility. In contrast, under white light, *Rpso* produced colonies with smooth margins that are not characteristic of this motility (Fig. 2c). In the case of *Rps*oΔ*lov,* the typical irregular projections of twitching motility were not observed in both lighting conditions.

### White light affects in vitro adhesion

**Figure 3﻿:**
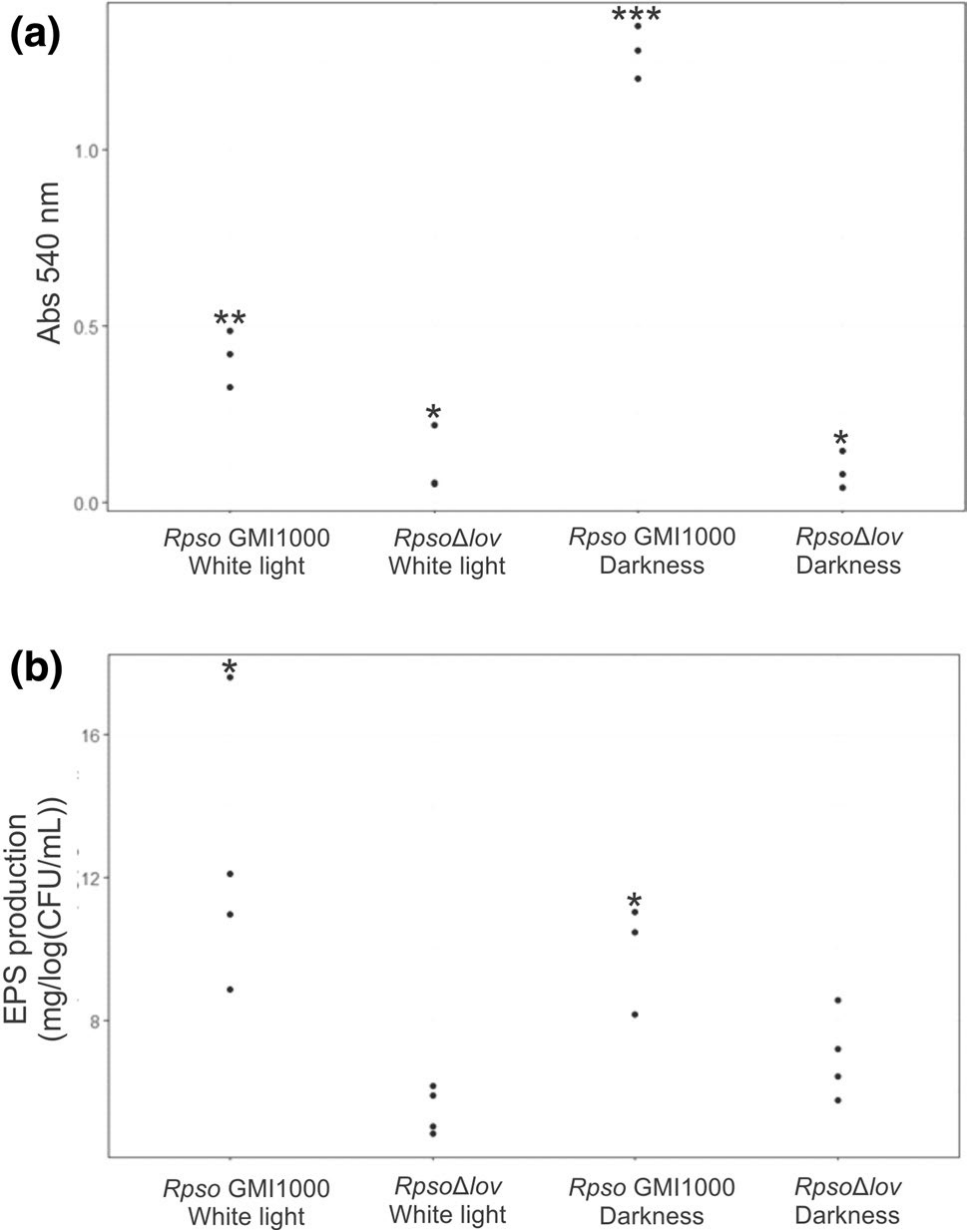
Evaluation of the adhesion of an abiotic surface and EPS production of *Rpso* strains. (**a**) Adhesion test of *Rpso* strains on abiotic surface. *Rpso* GMI1000 and *Rpso*Δ*lov* bacterial cells were grown under different lighting conditions adhering to the surface of the polystyrene plates and were stained with 0.1% (w/v) Crystal violet. Quantification diagram of the bound dye solubilized with ethanol measuring the absorbance at 540 nm. Significant differences between conditions are represented by different asterisks (**-*p < 0.0059, ***-*p < 0.00001 and **-***p < 0.00001). (**b**) Quantification of the EPS production of the *Rpso* GMI1000 strain and the strain with the deleted *Rpsolov* gene. EPS was extracted from bacterial supernatants and quantified after 2 days of growth under different lighting conditions. The weight of EPS was normalized to the log (CFU/mL) of culture. The asterisks (*) indicate that there is a significant difference between the corresponding data (p = 0.0007).

After invasion of the intercellular spaces, cells of *R. solanacearum* attach to the surfaces of plant cells as an initial step of host colonization and infection^44^. We study the binding capacity of *Rpso* GMI1000 and *Rps*oΔ*lov* strain to an abiotic surface under white light and darkness (Fig. 3a). As shown *Rpso* exhibited increased adhesion ability when it was incubated in darkness compared to white light (p < 0.00001). However, the mutant strain showed decreased ability to adhere to the surface with respect to the wild type strain under both conditions tested (p < 0.0059 and p < 0.00001).

### In vitro production of *Rpso* extracellular polysaccharides depends on the *Rpsolov* gene

*Ralstonia solanacearum* generates an extracellular polysaccharide (EPS) composed of a complex polymer of N-acetylated sugars. EPS is an important virulence factor during bacterial wilt, being responsible for clogging the vessels of the xylem and triggering symptoms^45^. To determine the effect of light on EPS production in both *Rpso* strains, we quantified the precipitated EPS from the two-day *Rpso* GMI1000 and *Rpso*Δ*lov* cultures grown under two different lighting conditions. No differences were observed in the production of EPS of the *Rpso* GMI1000 strain in white light and continuous darkness, but the results showed that in the absence of the *Rpsolov* gene there was a marked decrease in the generation of EPS in both lighting conditions (p = 0.0007) (Fig. 3b).

### White light modifies the biofilm formation in *Rpso*

**Figure 4:**
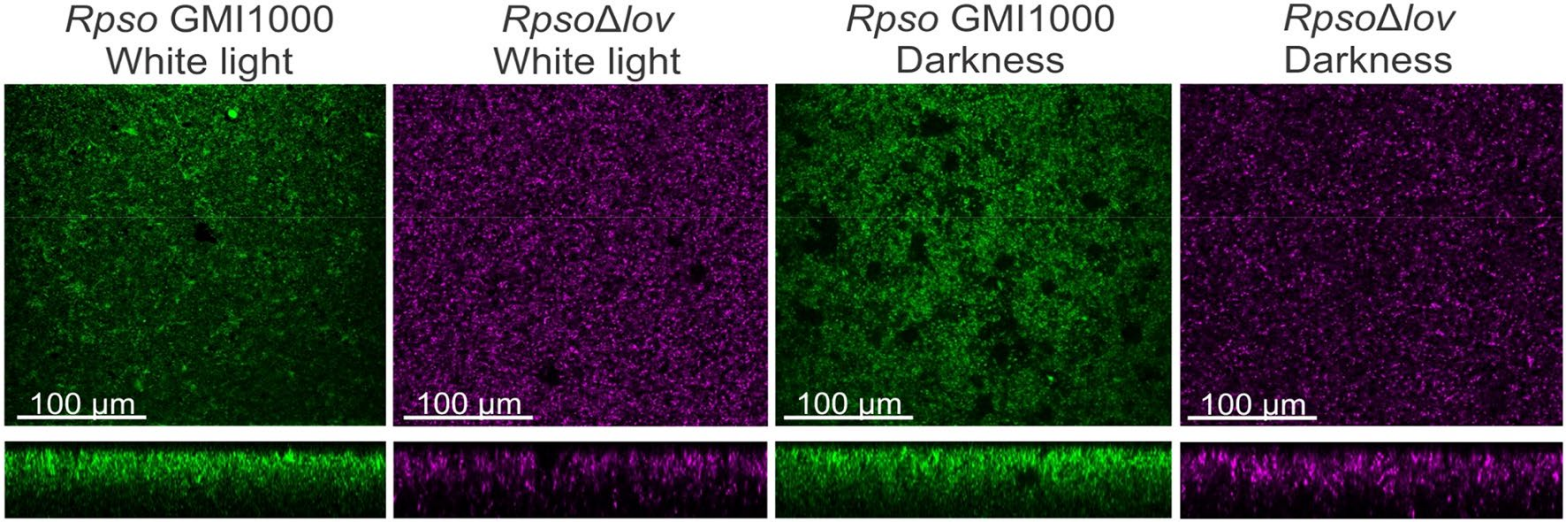
Biofilm architecture of *Rpso* strains under different lighting conditions. Confocal laser scanning microscopy images showing orthogonal views of biofilm formed by GFP and mCherry labeled wild type* Rpso * and *Rpso*Δ*lov* cultures after 3 days of static incubation in flat-bottomed microplates. Images are representative of results from three biological replicates (scale bar in inset, 100 µm).

We analyzed the morphology of bacterial biofilms developed by a GFP-labeled *Rpso* strain GMI1000 and mCherry-labeled *Rps*oΔ*lov* by confocal laser scanning microscopy (CLSM, Confocal Nikon C1SiR attached to a Nikon TE2000 inverted microscope)^46^. After three days of incubation under the different lighting treatments, *Rpso* GMI1000 generated a biofilm with a more structured, packed and organized topology, forming different layers in the dark than in white light. Bacteria appeared more dispersed in the last condition, similar to the mutant strain in both lighting conditions. In addition, a clear difference between the thickness of the biofilm of the wild strain with respect to the mutant strain was evidenced, the latter being thinner (Fig. 4).

### Expression analysis of the *Rpsolov* gene

**Figure 5:**
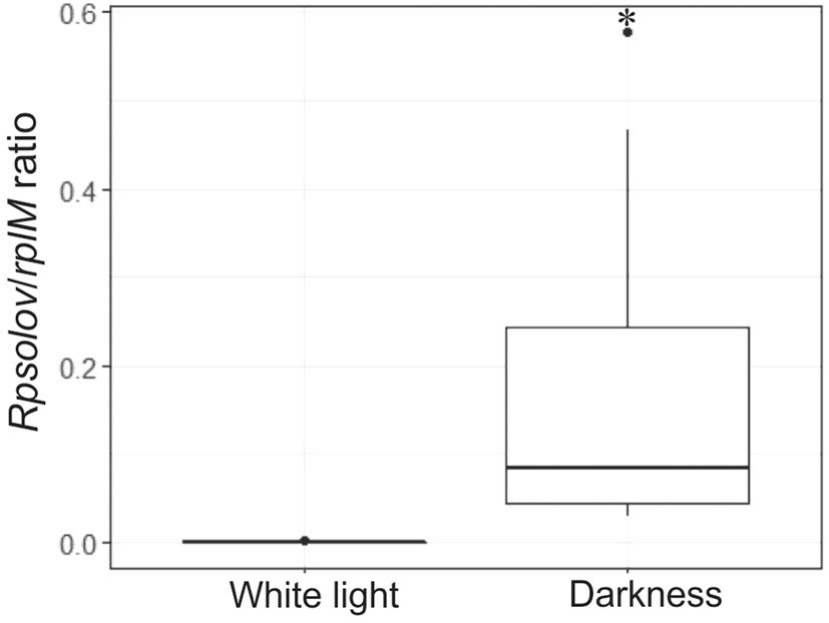
Gene expression analysis by quantitative real-time RT-PCR. The expression of the *Rpsolov* gene was assayed by RT-qPCR in *Rpso* GMI1000 under dark and light conditions using specific primers. Cultures grown for 18 h in MM medium were harvested to extract total RNA. The data shown report the relationship between the *Rpsolov* gene (*Rsp0254*) and the *rplM* reference gene in both lighting conditions tested. A significantly higher expression level was observed in the dark compared to white light. Asterisks (*) indicate significant differences between the corresponding data (p = 0.0004).

To analyze the *Rpsolov* gene expression, a reverse-transcription quantitative PCR (RT-qPCR) was performed with *Rpso* GMI1000 cultures grown under white light and darkness for 18 h in MM medium at 28 °C. *Rpsolov* gene was expressed in both lighting conditions, however a significantly higher level of expression was observed in darkness compared to white light (p = 4.114e−05) (Fig. 5). Experimental raw data in the software StepOne are shown in Supplementary material 3.

### Different transcriptional regulators control *Rpsolov* gene expression in *Rpso*

**Figure 6:**
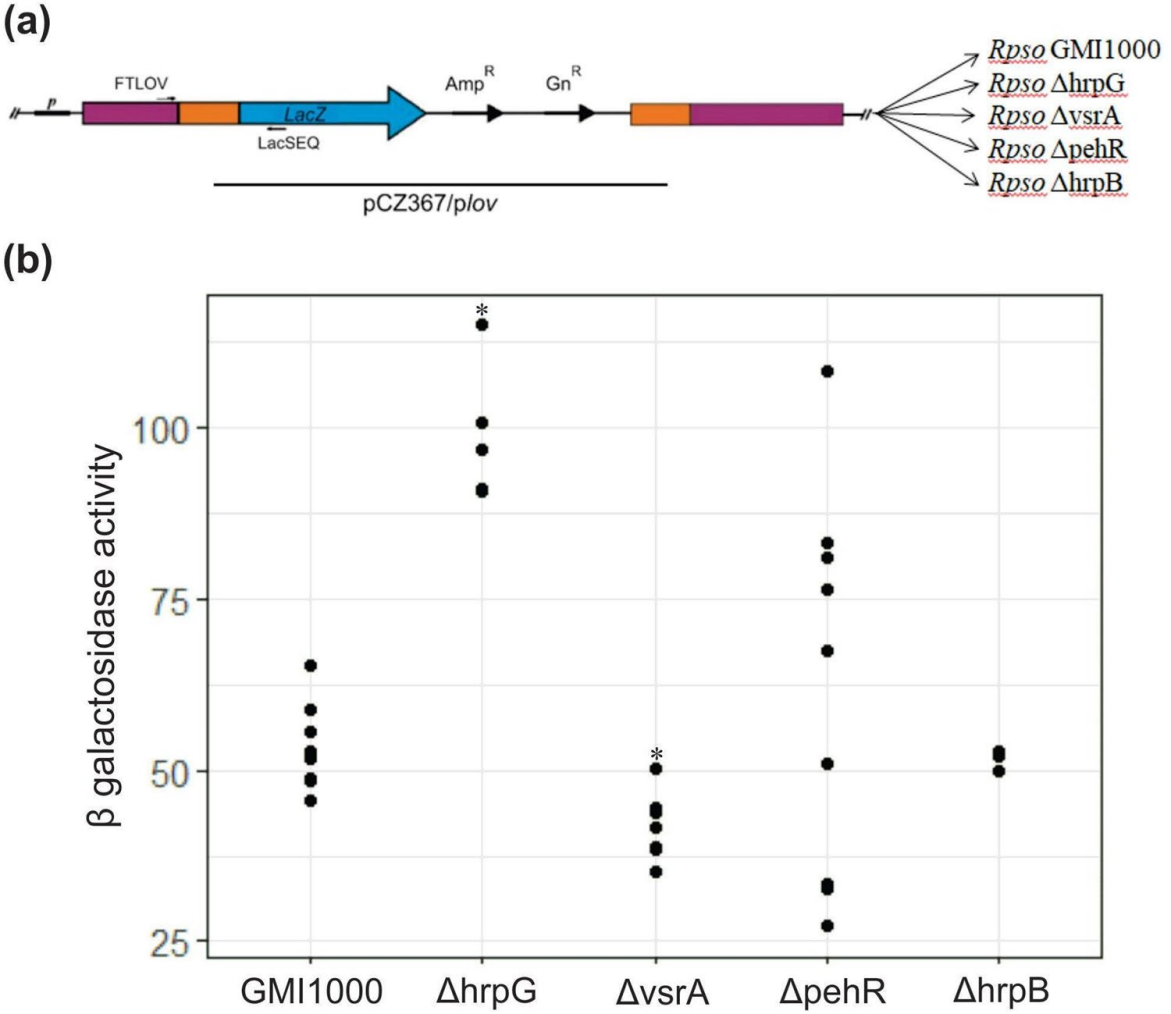
Expression of *Rpsolov* gene in different genetic backgrounds. (**a**) Schematic representation of the transcriptional fusion of the *Rpsolov* gene promoter with the *lacZ* gene in the different genetic backgrounds. (**b**) *Rpso* reporter strains were grown for 16 h in BG medium, β-galactosidase activity was measured and expressed in Miller units. The asterisks (*) in the dot plot indicate significant differences between the wild type strain and the Δ*hprG* (p < 0.001) and Δ*vsrA* (p = 0.0007) strains, respectively.

To determine if the *Rpsolov* gene expression is modulated during the infection process, we analyzed the transcriptional regulation of this gene. For that purpose, transcriptional fusions were generated between the promoter region of the *Rpsolov* gene and *lacZ*, which encodes the β-galactosidase enzyme (*lov*::*lacZ*). Then, these constructions were introduced into mutant strains for different transcriptional regulators that are known to regulate virulence genes in *Rpso.* Figure 6 shows the level of β-galactosidase activity monitored for each reporter strain. According to these results *Rpsolov* expression is negatively modulated by HrpG as the β-galactosidase activity of the *lov::lacZ* fusion is increased by a ~ twofold factor in the *hrpG* mutant strain in comparison to its expression in the wild-type (p ≤ 0.001). On the other hand, β-galactosidase activity levels were comparable in the wild type strain and in the *hrpB* mutant background.

In addition, an effect on the *lov::lacZ* expression was observed for the *vsrA* mutant, which exhibited significantly reduced β-galactosidase activity compared to the wild type strain (p = 0.0007).

In the case of the *pehR* and *hrpB* mutant strains, there were no significant differences in the activity measures with respect to the wild type strain.

### Environmental light quality defines the successful colonization of the host plant

The virulence of wilt type and mutant *Rpso* strains grown in white light and darkness conditions was tested in susceptible tomato plants by inoculation with the *Rpso* GMI1000 Pps-GFP reporter strain and *Rpso*Δ*lov* mCherry. The plants were kept in a normal photoperiod camera. 6 days after inoculation, before symptoms appeared, confocal laser scanning microscopy CLSM (Confocal Nikon C1SiR attached to a Nikon TE2000 inverted microscope) verified bacterial colonization in sections of root and plant stems.

**Figure 7:**
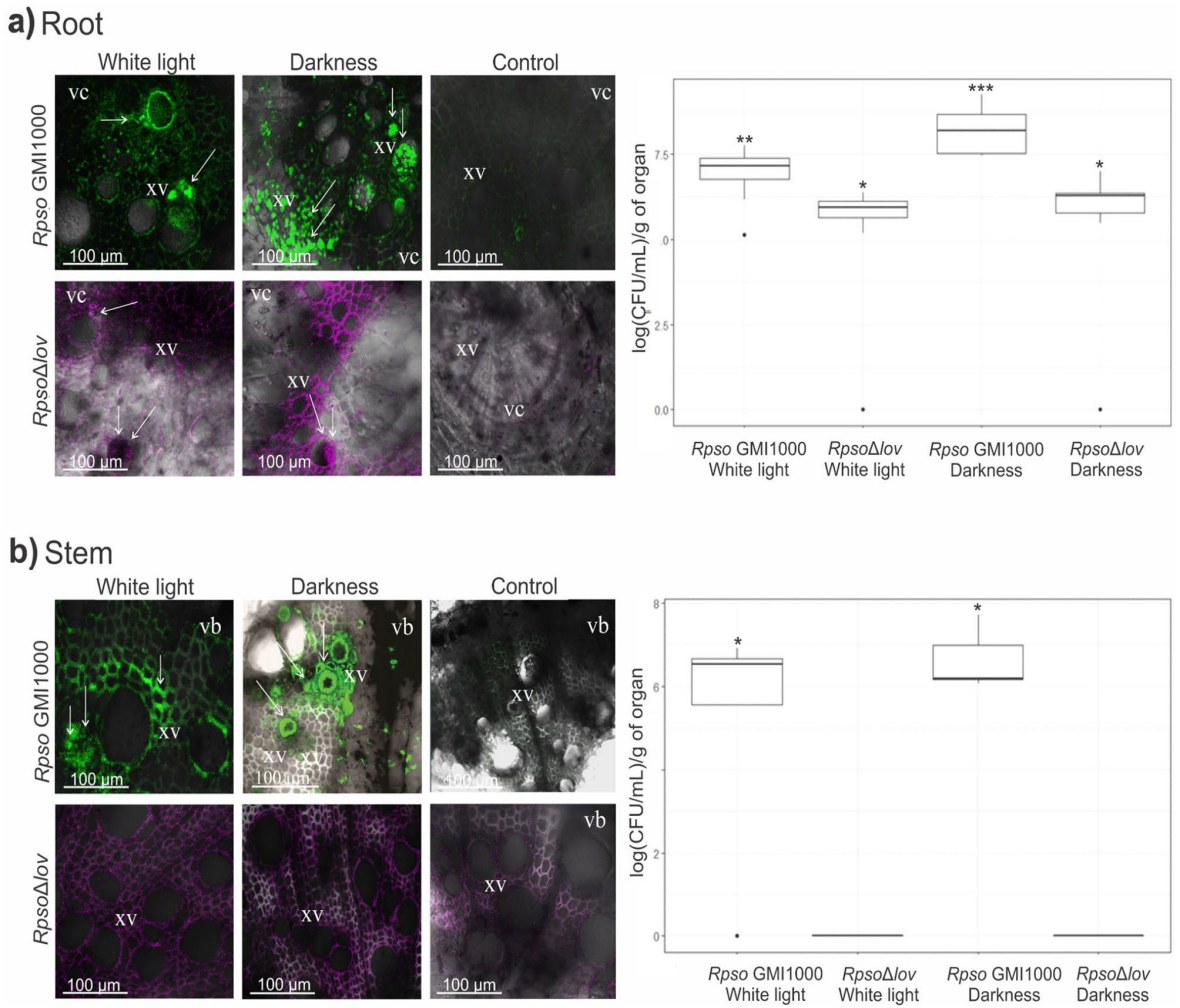
Roots and stems tomato plants colonization by *Rpso* and *Rpso*Δ*lov*. (**a**) Root cross sections observed by confocal laser microscopy indicating the presence of *Rpso* GMI1000 Pps-GFP and *Rpso*Δ*lov* mCherry previously grown under different lighting conditions. (**b**) Stem cross sections observed by confocal laser microscopy indicating the presence of *Rpso* GMI1000 Pps-GFP and *Rpso*Δ*lov* mCherry previously grown under different light conditions. The white arrows indicate the presence of pathogenic bacteria in the xylem vessels (xv) and other tissues present in the roots and stems of tomatoes. Each micrograph is a representative result of at least 10 sections of plant tissue from three biological replicates. Box plots of the bacterial population are shown under the different treatments in roots and stems of tomato plants at 6 dpi, respectively. Serial dilutions of the root and stem extracts were seeded on Rpso selective medium. The results were expressed as Log CFU/mL per gram of organ. The significant differences between the conditions are represented by different asterisks in the count of bacteria grown in both conditions in root and stem (root: **-*p < 0.00001, ***-*p < 0.00001 and **-***p = 0.0051536; stem: *p < 0.00001). xv, xylem vessels; vc, vascular cylinder; vb, vascular bundles.

It was observed that the analyzed strains were able to colonize the root system, noting an exacerbated invasion of the xylem vessels in tomato plants inoculated with *Rpso* GMI1000 cultures grown in darkness compared to plants that were inoculated with *Rpso* GMI1000 grown in light (Fig. 7a). On the other hand, the *Rpso*Δ*lov* strain grown in both lighting conditions invades in less quantity the root system than the WT strain, being observed dispersed throughout the tissue and colonizing some xylem vessels (Fig. 7a). Stem colonization with *Rpso*Δ*lov* is not observed (Fig. 7b). These representative images are supported by counting the CFU of bacteria obtained from root samples for the quantitative analysis of *Rps*o colonization. A greater number of bacteria were recovered from tomato roots inoculated with *Rpso* GMI1000 grown in white and dark light, the latter showing a greater difference (p = 0.0051536). The bacterial count of roots inoculated with the *Rpso*Δ*lov* strain grown in both lighting conditions was lower than the growth of the WT strain under the same conditions (p < 0.00001). There was no statistical difference in root growth for the *Rpso*Δ*lov* strain between both conditions (Fig. 7a).

On the other hand, it was observed that only the *Rpso* GMI1000 strain colonized the aerial part of the plant, showing a greater invasion tendency in the transversal sections of stems inoculated with the wild type strain grown in darkness. The *Rpso*Δl*ov* strain lost the ability to ascend and colonize plant stems. These representative images are consistent with the CFU count of bacteria obtained from tomato stems supporting quantitatively the observations provided by microscopy analysis (p < 0.00001) (Fig. 7b).

## Discussion

Environmental light is fundamental for the evolution and adaptation of all living organisms. Plants have developed abilities to maximize the capture of energy in their tissues and thus promote their development^47^. Until recently, light-induced signaling through photosensory proteins was considered an exclusive feature of photoautotrophic organisms. However, genome sequencing revealed the presence of photoreceptors in all life kingdoms^48^. The photoreceptor proteins identified in the genomes of several microorganisms, fungi, insects and plants suggest that the role of light goes far beyond the photosynthesis process. In the case of plants, light is not only essential for their survival but also to reinforce defense against pathogens^3^.

In phytopathogenic bacteria, a variety of photoreceptor proteins have been reported^9,49^. These proteins detect light to regulate various cellular processes such as motility, adhesion, morphology, multiplication, DNA repair, secondary metabolite production and bacterial colonization. Oberpichler et al*.* have provided evidence linking light perception and virulence through cell motility control in *Agrobacterium tumefaciens*^50^.

Previous studies have shown how light affects the plant-pathogen interaction, both regarding the host plant response and to the phytopathogen ability to infect the plant^5,7,9,16^. Several investigations have reported the influence of light on host and non-host plant interactions with Gram-negative bacteria: (1) biotrophic such as *Agrobacterium tumefaciens*^50^, (2) hemibiotrophic such as *X. citri* subsp. *citri*^9^, *Xanthomonas campestris* pv. *campestris*^51^ and *Pseudomonas syringae* pv. *tomato*^10^, (3) necrotrophic such as *Botrytis cinerea*^52^ and (4) endosymbionts such as *Rhizobium leguminosarum*^18^. In particular, the present work focuses on the study of the effect of light on interaction mechanisms between a vascular phytopathogen such as *Rpso* GMI1000 and tomato plants.

*Rpso* has a 5.8 Mbp genome formed by a chromosome and a megaplasmid. The megaplasmid genes analysis suggests that this replicon has a significant function in the bacterium adaptation to different environmental conditions^23^. A gene encoding a putative blue light photoreceptor (*Rpsolov* gene) was identified in this megaplasmid. Mandalari et al*.* studied in detail the organization of the LOV photoreceptor in the *Ralstonia* genus, mainly in *Rpso* GMI1000^33^. Through the application of bioinformatics programs determined that it would be a transmembrane protein composed by 1178 amino acids, with a di-guanylate cyclase domain fused to a phosphodiesterase domain as response regulator domain, unlike of those present in *Pseudomonas* and *Xanthomonas* genus that are cytosolic and histidine kinase or hybrid histidine kinase^9,10,53,54^. Many in silico analysis of LOV photoreceptor, such as multiple sequence analysis, indicated that the LOV domain and the response regulation domain were found in several members of the *Ralstonia solanacearum* species complex (Supplementary material S1). Similar results were observed in *Xanthomonas* genus^55^. The organization of hybrid LOV-HK-RR proteins were conserved almost exclusively in bacterial plant pathogenic species and they are involved in the regulation of different virulence factors at some stage of the bacterial life cycle through blue light sensing^9,56,57^.

In this work, the role of light and LOV protein in *Rpso* physiology and in the pathogenesis process was studied. For this purpose, the wild type strain *Rpso* GMI1000 and *Rps*oΔ*lov*, mutant strain due to complete deletion of the gene, were studied. In vitro growth curves of both strains were performed in white light and darkness (Fig. 1), observing that there are no differences in the bacterial numbers (CFU/mL) under the different light conditions, which indicates that the absence of *Rpsolov* gene and the light does not affect the bacterial viability and growth kinetics. These results agree with those described by Wu et al*.* and Kraiselburd et al., where the viability of *Pseudomonas syringae* pv. *syringae* and *X. citri* respectively was not affected by the different lighting conditions and *Rpsolov* gene deletion^9,16^.

Several reports show that light regulates the bacterial transition between a mobile and a sessile state^7,8^. *Rpso* moves towards a plant host when it perceives a stimulus or is attracted by the root exudates. We evaluated the effect of different lighting conditions on swimming and twitching motilities. Swimming motility is an individual translocation dependent on flagella that occurs in liquid media, water content being a critical factor for this displacement. As it is shown in Fig. 2a,b, the *Rpso* GMI1000 strain showed a greater displacement in darkness compared to white light, that is, white light inhibits swimming motility. In addition, it was observed that *Rpso*Δ*lov* strain presented a lower displacement than wild type strain in both lighting conditions. In this context, the LOV protein would be involved in the regulation of motility. Similar results were obtained with *P. syringae* pv. *tomato* DC3000 where motility repression was observed, as with *Rpso*, under the same light condition, also a *Psto* DC3000 mutant in the *Rpsolov* gene showed decreased motility compared to the wild type strain in both light conditions, indicating that the LOV-HK photoreceptor positively regulates this type of motility^10^. Similar results were obtained by our group for the LOV protein of *Xcc* where motility was also modified in the mutant strain of the *lov* gene^9^. This behavior was also found in the phytopathogen *A. tumefaciens*, which has phytochrome type photoreceptors, observing that bacterial suspensions grown in white light showed less motility compared to dark-grown cultures^50^. In addition, in *Xanthomonas oryzae* pv. *oryzae* (*Xoo*) was observed that the complete deletion of the bacteriophytochrome gene (BphyP Knockout) rendered a similar behavior than *Rps*oΔ*lov*, the mutant strain produced reduced swimming motility in all lighting conditions^58^.

Twitching is a type of translocation present in a wide variety of bacteria, including the genus *Pseudomonas* and *Ralstonia*. This type of motility depends on the type IV pili extension and active retraction and the moisture availability in the culture medium. The type IV pili are formed by polymerization of pilin monomers. This structure is involved in different biological processes, including adhesion, biofilm formation and horizontal gene transfer^59^. In *Rpso* it was demonstrated that these appendices are essential for pathogenicity^60^. Pilin is post-translational modified by glycosylation in Gram-negative bacteria and it has been reported that defective mutant strains in the pili production or glycosylation did not show contraction motility and caused reduced symptoms and slower disease progression. For example, in *Rpso* GMI1000, a mutant strain deficient in the gene that codes for an enzyme involved in the O-glycosylation of type IV pili, did not generate bacterial wilt symptoms when it was inoculated in tomato plants^61^. In our study when *Rpso* was grown under white light this motility was reduced, while, under dark conditions, the bacterium migrated via twitching. In absence of light, as can be seen in Fig. 2c, it was observed that colonies present irregular aspects and long bacterial extensions (raft) irradiated from the migration zones. Furthermore, in the presence of light, bacteria developed colonies with smooth edges, without visible bacterial extensions, suggesting that light inhibits *Rpso* capability to migrate via twitching motility. These results agree with those published by Bitrian^62^ and Hoff^63^ where light implication in the bacterial physiology of the environmental pathogen *Acinetobacter baylyi* was found. This bacterium has a blue light photoreceptor, BLUF type, responsible for this behavior. The evaluation of twitching motility in *Rpso*Δ*lov* allowed us to observe a lower displacement both in white light and in darkness, compared to the wild type strain (Fig. 2c). These results suggest that *Rpso* LOV protein is an activator of twitching motility in the assay conditions. This behavior was also observed in the *Rpsolov* mutant of *X*. *citri* subsp. *citri* which presented colonies with smooth borders different from the starry edges of the wild type strain^9^.

Several factors contribute to bacterial adhesion on the host tissue, including fimbrial and non-fimbrial adhesins, extracellular polysaccharides (EPS) and flagella^64,65^. In this work, *Rpso* GMI1000 ability to adhere to abiotic surfaces under different lighting conditions was evaluated. A drastic decrease in adhesion under white light (Fig. 3a) was observed, indicating that it is a light-dependent process. This result differs from the obtained by Río Alvarez et al. where differences in the adhesion capacity of the wild *Psto* DC3000 strain on *A. thaliana* leaves were observed. Moreover, in dark conditions or under red light the wild type strain did not adhere to the leaf surface after 6 h of incubation. However, when bacteria were pre-treated for 10 min with red light and then incubated for 6 h in the dark, they recovered the ability to adhere to *A. thaliana* leaves. These differences can be attributed to other photoreceptors present in *Psto* beside LOV type photoreceptor^10^. On the other hand, *Rpso* has only one putative photoreceptor that would sense the light absence in the soil depth allowing the capture of a host plant signal, root adhesion and then to initiate plant colonization. In the evaluation of *Rpso*Δ*lov* adhesion to abiotic surfaces, this strain lost the ability to adhere in all light conditions, this type of mutation generated by complete deletion of the gene and the observed phenotype allowed us to conclude that the LOV protein has a role as a positive regulator of adhesion in *Rpso* independently of light. These results agree with Kraiselburd et al. where the *X. citri* subsp. *citri* mutant in the *lov* gene presented in vitro and in vivo adhesion significantly diminished compared to the wild type, showing a strong dependence on light during bacterial growth^9^. *Caulobacter crescentus* is a Gram-negative bacterium widely distributed in soils, lakes and water of sea which plays a very important role in the carbon cycle. The genome of *C. crescentus* contains an operon that codes for a LOV-histidine-kinase protein (LovK) and a single domain response regulator (LovR) which interacts with LovK^66^. Studies by Purcell et al. revealed that a mutant in LovR of *C. crescentus* presented a severe loss in adhesion capacity compared to the wild type strain, indicating that this protein is also an adhesion positive regulator as LOV protein of *Rpso*^66^.

EPS is the main *Rpso* virulence factor that causes wilting by restricting the flow of water through the xylem vessels^67^ and also notably improves the speed and extent of stem colonization^68^. We analyzed the EPS content in minimal and rich media. In minimal medium the EPS production was similar in both bacterial strains and in all conditions tested (Supplementary material S2). In CPG rich medium no significant differences in EPS production was observed under the different lighting conditions in the *Rpso* GMI1000 strain. On the contrary, a marked decrease in EPS synthesis was observed for the *Rpso*Δ*lov* strain with respect to the wild strain (Fig. 3a). These results suggest that EPS production in *Rpso* GMI1000 could be regulated by the LOV protein acting as a light-independent positive regulator of exopolysaccharide synthesis. Similar results were observed for *X. citri* subsp. *citr*i, where light does not affect xanthan production under the lighting conditions tested^9^. This apparent absence of light regulation in the case of the wild strain is contrary to the expected results considering that the *Rpsolov* gene encodes a photoreceptor, but it has been shown that the activity of some LOV-type bacterial photoreceptors is modulated by other stimuli such as for example, the cytosolic redox state in conjunction with light and that they would also perceive not only blue light, but also red light. Bonomi et al. determined that one of the virulence factors regulated by the LOV-HK photoreceptor of *Rhizobium leguminosarum* is the production of EPS. The mutant in the *lov* gene showed, as in *Rpso* GMI1000, a lower capacity for EPS synthesis compared to the wild strain, however, the regulation of polysaccharide production in *R. leguminosarum* occurs through light , LOV-HK being the sensor involved in this process^18^.

Bacteria develop dense communities associated with a surface known as biofilms, which are essential for their persistence^69^ and play an important role in the virulence of many pathogenic bacteria^70^. The morphological form in multicellular aggregates arises from the interaction of bacterial genetic makeup and environmental cues^71^.

Initially *Rpso* invades the intercellular spaces of the roots, attaches itself to plant cells and then spreads within them. Quorum sensing is activated at this stage, leading to the formation of fungus-like biofilms^72^, which are necessary for the pathogenicity of *Rpso*. The planktonic bacterial cells released from the biofilms can invade the xylem vessels, ascend through it and secrete virulence factors such as EPS in the stem, again forming a thick biofilm as a structural scaffold in the vascular bundles to cause water obstruction. and thus induce wilt symptoms^72,73^.

When biofilms formation and architecture were analyzed using CLSM, we found that the macrocolony biofilm generated by *Rpso* GMI1000 in dark was structured with several layers leading to folds formation, rendering a more compact and organized biofilm compared to white light, where a macrocolony biofilm covers the surface more loosely. This last characteristic is also presented in the mutant strain in the *Rpsolov* gene, which shows the same phenotype (Fig. 4).

Our results agree with those of Mussi et al., where the opportunistic pathogen *Acinetobacter baumanni* develops a differential production of biofilm with a greater capacity to form biofilms in dark conditions^74^. In conclusion, the absence of light regulates the formation of biofilms in *Rpso* GMI1000 and *A. baumanni.*

On the other hand, we discovered a marked variability in the thickness of the biofilm structures between the two strains studied. The wild type strain was characterized by developing a thick biofilm with appreciable density, while *Rpso*Δ*lov* was thin and dispersed, concluding that the LOV protein is involved in the biofilm formation.

In view of the results observed in different types of motilities, biofilm formation and abiotic adhesion, we infer that light would be behaving as an inhibitor of the different virulence factors mentioned above, but when deleting the *Rpsolov* gene it was observed that this protein would act as a positive regulator of virulence features. *Rpso* GMI1000 has a single encoded photoreceptor protein in its genome that exhibits multiple domains as described above. In this work, a mutant strain was constructed in the complete gene (Knockout gene), without observing a phenotype that validates this hypothesis its role as a photoreceptor but that corroborates its participation in the regulation of the modified attributes in *Rpso*. Site directed mutants of *Rpsolov* gene site in other domains could provide a clearer role for this gene, since the phenotypes obtained could be associated not only with the LOV domain but also with other domains of this gene, such as the response regulatory domain^58^.

*Rpso* virulence was examined in tomato host plants 6 days after inoculation with wild-type *Rpso* and *Rpso*Δ*lov* grown under white or dark light conditions.

The wild type strain showed greater colonization of tomato roots and xylem vessels of stems inoculated with *Rpso* GMI1000 grown in the dark compared to those plants inoculated with bacteria grown in white light (Fig. 7a,b). Therefore, *Rpso* GMI1000 shows higher virulence in the dark condition. These results are consistent with the phenotype obtained with *Psto*, in a similar light treatment^10,11^. On the other hand, it was observed that *Rpso*Δ*lov* colonizes and disperses through the root system but loses the ability to ascend and multiply in the stem, showing that the deletion of the *Rpsolov* gene causes a decrease in virulence in the host plant (Fig. 7a,b).

Therefore, the bacterial physiological alterations caused by the light environment and the contribution of the *Rpso*l*ov* gene in the motility, adhesion and biofilm of *Rpso*, contribute to the successful propagation and colonization of roots and stems of the host plant.

In the case of the evaluation of *Rpsolov* gene expression in the two light conditions, real time quantitative analysis showed that in all conditions, the *Rpsolov* gene was expressed. The *Rpsolov*/*rplm* gene expression ratio in darkness was significantly greater than the ratio in white light (Fig. 5). This result shows an induction of the expression of *Rpsolov* gene in the dark. The same result was observed in *Acinetobacter baumannii* ATCC 17978. When this strain was incubated at 24 °C in light and darkness, the expression of *blsA* gene encoding a BLUF photoreceptor was higher in dark condition, but at 37 °C no differences in the *blsA* gene expression level was observed. These results indicate that temperature could play a role in the expression of *blsA*^74^. In this context, the induction of the *Rpsolov* gene in the dark could also be influenced by other environmental factors such as temperature, pH or redox state, as has been seen in other cases^75–77^. Further investigation of the *Rpsolov* gene will be essential to shed clarity on this issue.

Considering the wide range of biological functions affected by various environmental conditions, many of which are perceived by photoreceptor proteins, and according to the results described above where a light regulation of the *Rso* pathogenicity was observed, we decided to examine and provide an overview of the implication of the *Rpsolov* gene in the *Rpso* GMI1000 virulence factor regulation cascade which is sensitive to internal metabolism and environment. For this purpose, a transcriptional fusion was generated between the *Rpsolov* gene promoter and *lacZ* gene in the wild type *Rpso* strain and in different transcriptional regulators mutant strains. β galactosidase activity measurements then performed indicated that the *Rpsolov* gene is part of this network. These results showed that HrpG negatively regulates the *Rpsolov* gene expression under in vitro culture conditions (Fig. 6). HrpG, a response regulator belonging to the OmpR family, was originally discovered by positively regulating the HrpB expression, which controls the T3SS and activates the synthesis of 3-hydroxy-oxindole, a compound related to quorum sensing in early stages of *Rpso* infection^29,78^. Transcriptomic studies with *Rpso* revealed that the complete HrpG regulon controls several genes in addition to those regulated by HrpB^59^. HrpG controls functions that promote the bacteria adaptation to life within the host, as well as some virulence factors^79^. Our results suggest in this case that *Rpsolov gene* expression is controlled by HrpG in a HrpB-independent manner. On the other hand, the VsrA transcriptional regulator positively controls the *Rpsolov* gene expression. All these assays were performed in in vitro conditions. Despite extensive knowledge about how these networks work in culture, there are very few reports of the processes that occur in vivo during pathogenesis^22^. Recently it was shown that the expression of some of these transcriptional regulators depend on the conditions where the bacteria were grown. Perrier et al*.* studied the expression of these *Rpso* regulators in a complete medium and *in planta* conditions^26^. They showed that virulence functions corresponding to the HrpB and HrpG regulons are repressed by PhcA in complete medium but are specifically activated *in planta*. These regulons represent a set of key genes required for *Rpso* pathogenesis. Furthermore, it was reported that the expression of *Rso* T3SS genes are still effective in the xylem. Taking into account that the experiments to define the regulation cascade in relation to the *Rpsolov* gene were carried out under in vitro conditions and that the regulation of HrpG presents a contrasting regulation in vitro and *in planta* conditions^26^, it is necessary to carry out more investigation to clarify the role of light and HrpG in vivo. Furthermore, we observed in the case of the *pehR* strain that there were no significant differences with respect to the wild type strain under the conditions tested. The *pehR* gene is strongly expressed at low cell densities because it controls early virulence factors and is also a positive regulator of the swimming-type motility cascade^31^. PehR regulates both in minimal medium and in the plant, the expression of *flhDC*, an open reading frame that encodes the main regulator of flagellar biosynthesis and bacterial motility^80^. Probably, the expression of these regulators and the participation of the *Rpsolov* gene dependent on environmental factors in the *Rpso* regulatory cascade will ensure the expression of genes related to virulence at the appropriate time.

**Figure 8:**
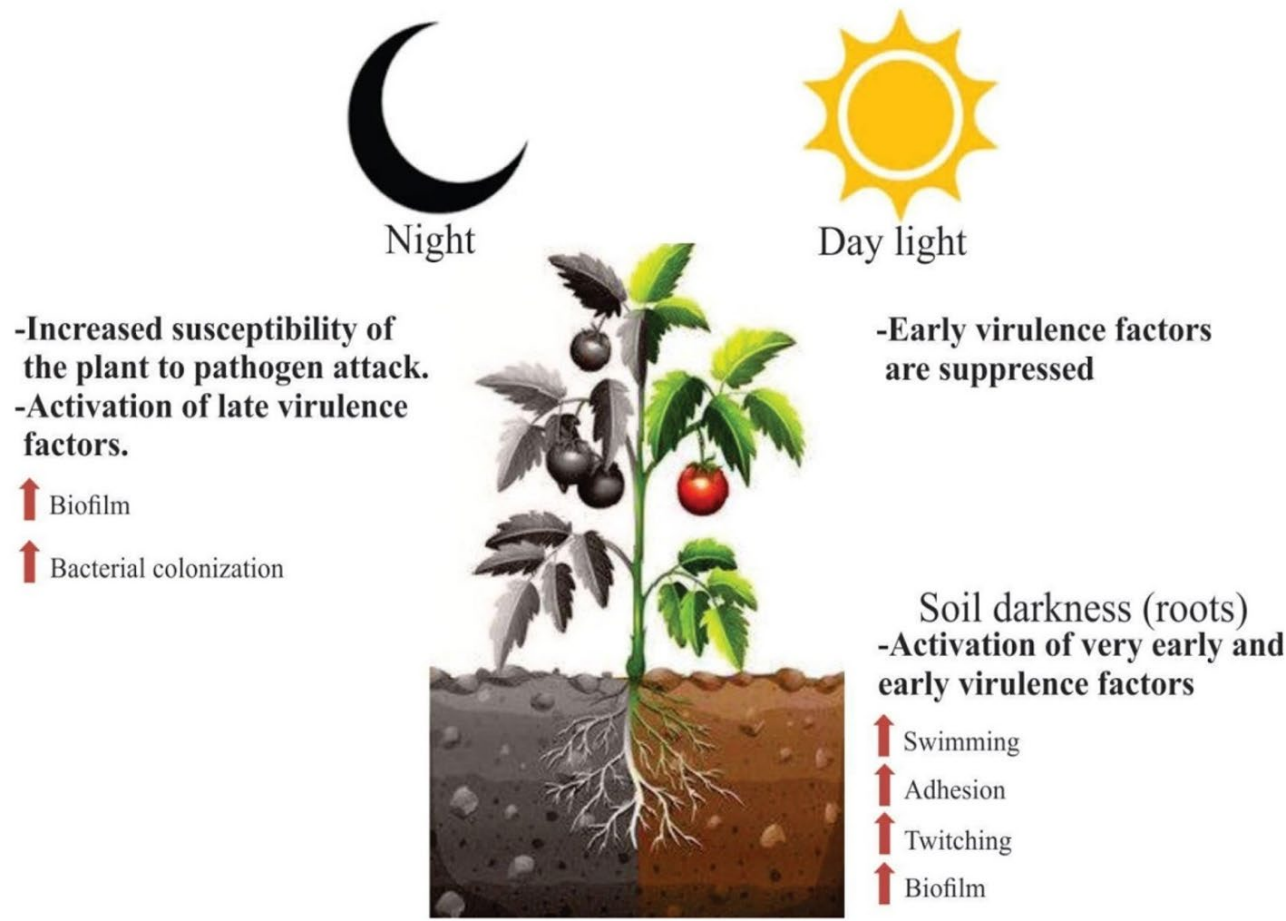
Integrative model illustrating *Rpso* GMI1000 light detection on virulence factors during interaction with tomato host plants. When the bacterium is in the ground (in the dark) and receives a specific stimulus from the host, the "very early virulence factors" are activated to reach, enter and colonize the roots through the "early virulence factors". Once inside the plant, propagation by xylem beams begins, detection of daylight begins and repression of "early virulence factors" occurs. During the night, again in the darkness, the bacterium detects the absence of light and takes advantage of the fact that the plant is more susceptible to attack by pathogens and, therefore, activates the "late virulence factors" that trigger bacterial wilt.

Finally, we have proposed a model to integrate the results obtained in *Rpso* physiological characterization and in the pathogenicity under the different lighting conditions (Fig. 8). Briefly, when bacteria are in the soil, in darkness, the so-called “very early and early virulence factors'' are activated, rendering a higher motility both swimming and twitching, greater adhesion and biofilm in the intercellular spaces of roots. Once *Rpso* enters the host plant, it invades the xylem vessels where it is capable to perceive the daylight in the aerial parts of the plant repressing the virulence factors, allowing the bacterium to raise the aerial host tissues and thus avoiding plant defense mechanisms. During the night, again in the darkness, the plant became more susceptible to biotic stress^3^. Under this situation, the bacterium activates the late virulence factors, producing more biofilm and allowing greater plant colonization (Fig. 8). These modifications in the bacterial behavior agree with the variation in the gene profile expression of *R. solanacearum* detected by RNAseq analysis using bacteria isolated from different regions of the plant tissues^27,81,82^.

It can be concluded that light act regulating several *Rpso* features directly involved in the pathogenicity process allowing a successful host colonization and infection. Furthermore, the *Rpsolov* gene and light would act as an essential bacterial factor that indicates position in the host plant, to regulate expression of virulence genes. Consequently, bacteria use an external signal and the LOV protein to know their location within plant tissue during the colonization process. Since *Rpsolov* gene presents a diguanylate cyclase and a phosphodiesterase C-terminal domain as a response regulator, the phenotypes observed for mutant bacteria could be associated with the pleiotropic effect modulated by a second messenger c-di-GMP. Further investigation of the putative blue light photoreceptor encoded by *Rpsolov* gene, will be essential to shed light into this question.

In summary, in this work is presented for the first time the role of light in the lifestyle of *R. pseudosolanacearum*, a vascular phytopathogen, demonstrating that the quality of this factor enables successful interaction with the host plant*.*

## Materials and methods

### Plasmids, bacterial strains and growth conditions

Bacterial strains and plasmids used in this study are listed in Table 1. *Rpso* cells were cultured in different media, mainly in Bacto-glucose (BG) medium or BG-1.5% (w/v) agar supplemented with 0.005% (w/v) tetrazolium chloride and 0.5% (w/v) glucose^83^. Alternatively, Casaminoacids-Peptone-Glucose (CPG) medium^84^, Boucher’s minimal medium (MM) supplemented with 20 mM l-glutamate as a carbon source^85^ or semi-selective SMSA medium (mSMSA) supplemented with 25 mg/L Bacitracin, 100 mg/L Polymyxin B sulphate, 5 mg/L Chloramphenicol, 0.5 mg/L Penicillin-G, 5 mg/L Crystal violet, 1 mg/L Cicloheximide and 50 mg/L 2,3,5-triphenyl tetrazolium chloride were used for *Rpso* growth^86^. *Escherichia coli* JM109 used for genetic constructions was cultured at 37 °C in Luria–Bertani medium^87,88^. For selection of the reporter strains gentamicin, 5 and 10 µg/mL was used in liquid and solid media, respectively.

**Table 1:** Bacterial strains and plasmids.

Bacterial strains/plasmids	Relevant characteristics	References
***Escherichia coli***		
JM109	e14-(MCRA-), *rec*A1, *hsd*R17, *end*A1, *thi*, *gyr*A96, *rel*A1, *sup*E44, Δ(*lac*-*pro*AB)/F’ [*tra*D36, *pro*A + B + , *lac*I^q^, *lac*Z ΔM15]	^87^
***Ralstonia solanacearum***		
*R. pseudosolanacearum* GMI1000	Wild type strain, Phylotype I, Origin: French Guyana	^23,88,89^
*R. pseudosolanacearum* Δ*lov*	*lov* mutant of *Rspo* GMI1000, Gm^r^	This work
GMI1000 Pps-GFP	*Pep*::*GFP*, Gm^r^	^46^
*Rpso*Δ*lov*::*mCherry*	*Pbb2::mcherry,*Gm^r^ Kn^r^	This work
Δ*hrpG*	*hrpG* deletion mutant in the GMI1000 background	^90^
Δ*vsrA*	vsrA::Ω, Sp^r^	Personal collection Stephane Genin
Δ*pehR*	pehR::Ω, Sp^r^	^91^
Δ*hrpB*	hrpB::Ω, Sp^r^	^92,93^
GMI1000/*lov*::*LacZ*	*lov*::*LacZ*, Gm^r^	This work
Δ*hrpG*/*lov*::*LacZ*	*lov*::*LacZ*, Gm^r^	This work
Δ*pehR*/*lov*::*LacZ*	*lov*::*LacZ*, Gm^r^	This work
Δ*vsrA*/*lov*::*LacZ*	*lov*::*LacZ*, Gm^r^	This work
Δ*hrpB*/*lov*::*LacZ*	*lov*::*LacZ*, Gm^r^	This work
**Plasmids**		
pCZ367	Insertional vector with *lacZ* reporter, Amp^r^, Gm^r^	^94^
pGEM-T easy	Cloning vector, Amp^r^	Promega
pCM351	Gm^r^, Amp^r^, Tm^r^; twoo SCM; allelic exchange vector	^95^
PB2-mCherry	Kn^r^	^96^Provided by Dr. Eleonora García Véscovi from her personal collection

Physiological assays were performed under different lighting conditions. For light condition, bacteria were grown in a chamber with continuous white light (130 μmol/m^2^s) provided by LEDs. For dark conditions, flasks or plates were covered with aluminum foil.

### Construction of the *Rpso*Δ*lov* mutant strain

To study the possible participation of the *Rpso* LOV protein in bacterial physiology, a mutant strain in the *Rsp0254* gene was constructed. For this, the gene was replaced by a Gm resistance cassette present in the suicide vector pCM351^95^. The upstream and downstream regions to the *Rsp0254* gene were amplified by PCR using the primers *Rpso*DOWNLOV-Fw (5′-GTTAACGCGCGCTTCACGGTGTAG-3′), *Rpso*DOWNLOV-Rv (5′-GAGCTCGACTGGCTGTGGCTCACC-3′), *Rpso*UPLOV-Fw (5′-GGAATTCCTGGCCCGACGATATAG-3′) and *Rpso*UPLOV-Rv (5′-GGGGTACCTTGGATGACCGGTAGAGCC-3′). The fragments were cloned on pCM35194 using the corresponding restriction sites. *Rpso* cells were transformed with the recombinant plasmid by natural transformation. The mutant strain was obtained by integration of the cloned fragment into the megaplasmid through a double homologous recombination event and selected by gentamicin resistance.

### Growth curves in different lighting conditions

Saturated cultures of *Rpso* GMI1000 and *Rpso*Δ*lov* grown in the darkness were sub-cultivated at 1% inoculum in BG fresh medium and incubated under white light or darkness conditions at 28 °C with shaking at 200 rpm. In order to determine the colony forming units (CFU)/mL, aliquots of cell suspensions were taken at different times. Three biological replicates in each lighting condition were used for the wild strain, while 2 were used for the mutant strain.

### Swimming assay

Overnight cultures of *Rpso* GMI1000 and *Rpso*Δ*lov* strains grown in darkness were washed with distilled water and adjusted to 10^7^ CFU/mL. Aliquots of 3 µL of these suspensions were inoculated on the center of BG-0.3% (w/v) agar plates and incubated at 28 °C under white light or darkness. The diameters of the swimming areas were measured at 48 h post-inoculation^97^. Six biological replicates were used in each condition tested.

### Twitching assay

Twitching motility tests were carried out following the protocol described by Siri et al*.*^98^. Petri dishes were prepared with CPG-1.6% (w/v) agar. Bacteria were grown overnight in darkness at 28° C in liquid CPG medium with shaking. The wild-type and mutant *Rpso* cultures were diluted to obtain a final concentration of 10^9^ CFU/mL, and 10 μL of the bacterial suspensions were then inoculated on the surface of the CPG plates. The plates were incubated in different lighting conditions at 28° C in a humid chamber for 24 h. Motility was examined by optical microscopy (Carl Zeiss, Axiostar, Germany), using a 20× objective.

### In vitro adhesion assay

In vitro adhesion of the studied strains was determined using polyvinyl chloride microtiter plates (Nunc MicroWell plate; Thermo Fisher Scientific Inc., Waltham, MA, USA). *Rpso* GMI1000 and *Rpso*Δ*lov* saturated cultures grown in MM medium were adjusted to 10^6^ CFU/mL and 100 µL of cell suspension were placed on said plates. Plates were incubated statically in different lighting conditions at 28 °C for 6 h. To quantify cell aggregation, 25 µL of 1% (w/v) Crystal violet solution was added to the wells. After 15 min incubation, unbound Crystal violet was gently removed with a pipette and the wells were washed with distilled water. Subsequently, 200 μL of 95% (v/v) ethanol were added and carefully resuspended the Crystal violet adhered to the cells. Bacterial adhesion was quantified by measuring the absorbance at 540 nm of the obtained solution^44^.

### Biofilm formation assay

Biofilm formation analyses were performed with a modified *Rpso* strain that constitutively expresses the green fluorescence protein (GFP)^46^ and a LOV protein mutant strain transformed with a plasmid overexpressing mCherry^96^. Saturated cultures *Rpso* grown in CPG medium in darkness were adjusted to 10^7^ CFU/mL, diluted 1∶20 in fresh medium and then 300 µL of the bacterial suspensions were placed into chamber covered glass slides (N°155411, Lab-Tek, NUNC, Naperville. IL, U.S.A.). Chambers were statically incubated in a humidified polyvinyl chloride (PVC)-box at 28 °C under the different light conditions. Biofilm formation was visualized by confocal laser scanning microscopy (CLSM,Confocal Nikon C1SiR attached to a Nikon TE2000 inverted microscope)^99^. Images obtained were analyzed with ImageJ software.

### EPS production

Quantification of EPS production by *Rpso* was performed following the protocol described by Peyraud et al. with some modifications^45^. *Rpso* GMI1000 and *Rpso*Δ*lov* saturated cultures grown in darkness were subcultured in 100 mL of minimal medium (MM) supplemented with 20 mM l-glutamate as a carbon source. Subsequently, they were incubated for 48 h at 28 °C in the two different lighting conditions. Aliquots of 5 mL of cell suspensions were filtered with 0.22 µm pore filters and the supernatants were collected. In order to precipitate the EPS, 20 mL of isopropanol and 0.36 mL of 0.3 M NaCl were added to the supernatants followed by the incubation at 4 °C for 72 h. Then, the mixtures were centrifuged at 4 °C for 10 min at 16,000*g* and the supernatants discarded. Pellets were dried for 15 min at room temperature and the dry weights determined. Subsequently, we made a modification in the way of obtaining bacterial suspensions where the saturated cultures of *Rpso* GMI1000 and *Rpso*Δ*lov* that grew in the dark were subcultured in 10 mL of medium rich in CPG. Then continued with protocol described by Peyraud et al^45^.

### RNA extraction, reverse transcription (RT), and quantitative real-time PCR (qPCR)

*Rspo* GMI1000 was cultured 18 h in MM medium under white light and darkness. Total RNA was isolated using TRIzol reagent (Invitrogen), according to the manufacturer's instructions. The extracted RNA was treated with RNase-free DNase (Promega) and its integrity was checked by agarose gel electrophoresis. For cDNA synthesis, total RNA (1 µg) was added to a 20 µl reverse transcription reaction medium containing 4 µl 5 × M-MLV buffer (Promega), 0.5 mM dNTP mixture, 0.5 µg random hexamer primer (Invitrogen), 200 U M-MLV reverse transcriptase (Promega) and incubated for 60 min at 42 °C. Reverse transcription was terminated by incubating for 5 min at 94 °C. qPCR was carried out using HOT FIREPol EvaGreen qPCR Mix Plus (Solis Biodyne), following the manufacturer's instructions. Primers RTlov-Fw (5′-TCAACATCGACCGCTTCAAG-3′) y RT2lov-RV (5′-AGCGCGAAGACGTCGCC-3′) were used for the *Rpsolov* gene and primers RTrplM-Fw (5′-GCGCAATTGGTTCGTGATTG-3′) y RT2-rplM-RV (5′-GGCTGCGTTGATCACGATG-3′) were used for constitutive control gene *rplM*. The StepOne Real-Time PCR system (Applied Biosystems) was used. qPCR reactions were carried out under the following conditions: initial denaturalization at 95 °C for 12 s, and 40 cycles of amplification at 95 °C for 15 s, annealing 60 °C for 25 s and extension at 72 °C for 20 s. Three biological replicates were analyzed three times. The amount of transcripts was presented as the ratio between the gene of interest and the reference gene (applying 2^−ΔCt^ where ΔCt refers to the difference in the threshold cycles between the genes of interest and reference).

### Generation of *Rpsolov* reporter strains

A transcriptional fusion of the *Rpsolov* gene promoter with the *lacZ* gene was generated by using integration plasmid pCZ367^94^. Briefly, a 1000 bp fragment containing the promoter region and the beginning of the coding sequence of *Rpsolov* gene was PCR amplified with primers LOVFT-Fw (5′ AAGCTTTCTCGTACGAAACCCAGAGC 3′) and LOVFT-Rv (5′ TCTAGAGTCAGGTGGTGGACGGTCT 3′) and cloned into the *Hind*III and *Xba*I sites of pCZ367. The resulting plasmid was then introduced into the different genetic backgrounds (GMI1000, Δ*hrpG,* Δ*pehR,* Δ*vsrA,* Δ*hrpB*) by electroporation (2.5 kV, 200 W, 25 µF, 0.2-cm cuvette gap) and the recombinant clones were selected by pCZ367 Gentamicin resistance. Integration of the vector in the correct site of the bacterial genome by a simple recombination event was checked by PCR using the primers UPLOVFT-Fw (5′CATGCTTTCTTTCCCACCAC3′) and Lacseq-Rv (5′TGTAAAACGACGGGATCCAT 3′), which hybridize upstream of the *Rspolov* fragment used for the recombination and in the *lacZ* gene, respectively. Measurements of β-galactosidase activity were performed as described by Brito et al.^100^. All these assays were realized without light treatment.

### Virulence assay

For pathogenicity tests, night cultures of the reporter strain *Rpso* GMI1000 Pps-GFP and *Rpso*Δ*lov* mCherry grown in dark and white light at 28 °C were adjusted to a concentration of 10^7^ CFU/mL. Tomato plants (*Solanum lycopersicum* var. Minitomato) were inoculated with 20 mL of the bacterial suspensions to achieve a final concentration of 10^6^ CFU/g^101^. The roots were injured before inoculation. Plants inoculated with sterile water were used as negative controls. To determine the amount of bacteria (CFU) at 6 days post-inoculation, the plants were disinfected with 70% ethanol (v/v) for 3 min, immersed in sterilized water for 3 min and dried with sterile absorbent paper. The roots and 1 cm sections of the stems were cut and weighed. Subsequently, both tissues were ground in sterile water and serial dilutions of the bacterial suspensions were streaked onto mSMSA plates and incubated 7 days at 28 °C. In addition, 10 cross-sections of the main root and stem of the control and inoculated plants were cut with a disinfected scalpel by hand and visualized by CLSM (Confocal Nikon C1SiR attached to a Nikon TE2000 inverted microscope) to analyze the bacterial colonization in stem and root xylem vessels^101^. Images obtained were analyzed with ImageJ software.

### Statistical analyses

Statistical analysis was performed with R statistical software (R Foundation for Statistical Computing, Vienna, Austria). To compare the growth curves, the data were analyzed using a mixed model of repeated measures (longitudinal data), considering three fixed factors, condition (at two levels: growth in white light and darkness), strain (*Rpso* GMI1000 and *Rpso*Δ*lov*) and time (at 10 levels) and culture as a random factor. It was considered significant when p < 0.05.

To fulfill the objective of the work, a non-parametric bifactorial ANOVA (two-way ANOVA) test was applied in the motility, adhesion, EPS production, and bacterial count in roots and stem tests. A p < 0.05 was considered statistically significant.

The comparison of continuous variables (ß-galactosidase activity and *Rpsolov* gene expression) in different subgroups was performed using the Mann–Whitney U test. The analysis was performed at a significance level of 5% and a p value less than 0.05 was considered statistically significant.

## Supplementary Information

Supplementary Information 1.

Supplementary Information 2.

Supplementary Information 3.
