# Supplementary material for: Light modulates important physiological features of Ralstonia pseudosolanacearum during the colonization of tomato plants
Source: Sci Rep. 2021 Jul 15;11:14531. doi: 10.1038/s41598-021-93871-9 (PMC8282871; doi:10.1038/s41598-021-93871-9)
Supplement: Supplementary file 1 — Supplementary Information 1. [file 41598_2021_93871_MOESM1_ESM.pdf]

Light modulates important physiological features of *Ralstonia pseudosolanacearum* during the colonization of tomato plants.

Josefina Tano[1‖], María Belén Ripa[1‖], María Laura Tondo[2], Analía Carrau[1], Silvana Petrocelli[2], María Victoria Rodriguez[3], Virginia Ferreira[4], María Inés Siri[4], Laura Piskulic[5], Elena Graciela Orellano[1*].

[1]Instituto de Biología Molecular y Celular de Rosario, Facultad de Ciencias Bioquímicas y Farmacéuticas, Universidad Nacional de Rosario, (IBR-CONICET-UNR), Rosario, Argentina.

[2]Facultad de Ciencias Bioquímicas y Farmacéuticas, Universidad Nacional de Rosario, Rosario, Argentina.

[3]Área Biología Vegetal (CONICET), Facultad de Ciencias Bioquímicas y Farmacéuticas, Universidad Nacional de Rosario, Rosario, Argentina.

[4]Área Microbiología, Departamento de Biociencias, Facultad de Química, Universidad de la República, Montevideo, Uruguay.

[5]Área estadística y procesamiento de datos, Facultad de Ciencias Bioquímicas y Farmacéuticas, Universidad Nacional de Rosario, Rosario, Argentina.

[‖] These authors contributed equally to the work.

*To whom correspondence should be addressed at: Elena G. Orellano, IBR-CONICET-UNR, FCByF-UNR, Suipacha 531, (S2002LRK) Rosario, Argentina. Phone: +54 341 4350661. FAX: +54 341 4390465. Email: orellano@ibr-conicet.gov.ar

Supplementary material S1. Multiple alignment of amino acid sequences of LOV proteins distributed in the *Ralstonia solanacearum* species complex (RSSC). LOV domains orthologous to the *Rpso* GMI1000 photoreceptor were searched using the BLAST 21 (Basic Local Alignment Search Tool-NCBI) network service, on all annotated genome sequences of the genus *R. solanacearum*. The threshold of the expected value was set at 0.0001 and the sequences were filtered for low complexity regions. Sequence alignments were performed using a PRELINE multiple sequence alignment tool. The least conserved sequences are shown in green to blue, while the sequences identified in orange to red are the most conserved.

Results colour-coded for amino acid conservation

The current colourscheme of the alignment is for **amino acid conservation**.

The conservation scoring is performed by PRALINE. The scoring scheme works from 0 for the least conserved alignment position, up to 10 for the most conserved alignment position.
The colour assignments are:

Unconserved 0 1 2 3 4 5 6 7 8 9 10 **Conserved**

```
                  ......... 10  ......... 20  ......... 30  ......... 40  ......... 50
tr_GMI1000__tr_   ----------    ----------    ----------    ----------    ----------
tr_OE1-1__AVV67   ----------    ----------    ----------    ----------    ----------
tr_CMR15__tr_D8   ----------    ----------    ----------    ----------    ----------
tr_FQY_4_tr_M4U   ----------    ----------    ----------    ----------    ----------
tr_CFBP2957__tr   ----------    ----------    ----------    ----------    ----------
tr_UYO31__tr_A0   ----------    ----------    ----------    ----------    ----------
tr_IPO1609__CEJ   ----------    ----------    ----------    ----------    ----------
tr_K60__OYQ0909   ----------    ----------    ----------    ----------    ----------
tr_R_syzygii_R2   ----------    ----------    ----------    ----------    ----------
tr_Po82__tr_F6G   MQAKCLRTAS    AVESAAVEPR    SHPRRAIEGR    RCRSRAGTAT    LDLDALPNTQ
tr_UW551__EAP72   MQAKCLRTAS    AVESAAVEPR    SHPRRAIEGR    GCRSRAGTAT    LDLDALPNTQ
tr_PSI07__CBJ34   ----------    ----------    ----------    ----------    ----------
Consistency       0000000000    0000000000    0000000000    0000000000    0000000000

                  ......... 60  ......... 70  ......... 80  ......... 90  ......... 100
tr_GMI1000__tr_   ------MPKH    AFFPTTLRGR    LLALAAVAAL    PAVVVAIAGV    ALYRDRLTDH
tr_OE1-1__AVV67   ------MPKH    AFFPTTLRGR    LLALAAVAAL    PAVVVAIAGV    ALYRDRLTDH
tr_CMR15__tr_D8   ------MPKH    AFFPTTLRGR    LLALAAVAAL    PAVVVAIAGV    ALYRDRLTDH
tr_FQY_4_tr_M4U   ------MPKH    AFFPTTLRGR    LLALAAVAAL    PAVVVAIAGV    ALYRDRLTDH
tr_CFBP2957__tr   ------MPKH    AFFPTTLRGR    LLALAAVAAL    PAVIVAIAGV    ALYRDRLTDH
tr_UYO31__tr_A0   ------MPKH    AFFPTTLRGR    LLALAAVAAL    PAVIVAIAGV    ALYRDRLTDH
tr_IPO1609__CEJ   ------MPKH    AFFPTTLRGR    LLALAAVAAL    PAVIVAIAGV    ALYRDRLTDH
tr_K60__OYQ0909   ------MPKH    AFFPTTLRGR    LLALAAVAAL    PAVIVAIAGV    ALYRDRLTDH
tr_R_syzygii_R2   ------MPKH    AFFPTTLRGR    LLALAAVAAL    PAVVVAIAGV    ALYRDRLTDH
tr_Po82__tr_F6G   ALPVIQVPKH    AFFPTTLRGR    LLALAAVAAL    PAVIVAIAGV    ALYRDRLTDH
tr_UW551__EAP72   ALPVIQVPKH    AFFPTTLRGR    LLALAAVAAL    PAVIVAIAGV    ALYRDRLTDH
tr_PSI07__CBJ34   ------MPKH    AFIPTTLRGR    LLALAAVAAL    PAVVVAIAGV    ALYRDRLTDH
Consistency       0000008***    **9*******    **********    ***9******    **********

                  ......... 110 ......... 120 ......... 130 ......... 140 ......... 150
tr_GMI1000__tr_   LEQRLSYETQ    SAAARVGMVL    SNADQLLSVV    VADESVLRLD    RDECTRFVSR
tr_OE1-1__AVV67   LEQRLSYETQ    SAAARVGMVL    SNADQLLSVV    VADESVLRLD    RDECTRFVSR
tr_CMR15__tr_D8   LEQRLSYETQ    SAAARVGMVL    SNADQLLSVV    VADESVLRLD    RDECTRFVSR
tr_FQY_4_tr_M4U   LEQRLSYETQ    SAAARVGMVL    SNADQLLSVV    VADEAVLRLD    RDECTRFVSR
tr_CFBP2957__tr   LEQRLSYETQ    SAAARVGMVL    SNADQLLSVV    VADESVLRLD    RDECTRFVGR
tr_UYO31__tr_A0   LEQRLSYETQ    SAAARVGMVL    SNADQLLSVV    VADESVLRLD    RDECTRFVGR
tr_IPO1609__CEJ   LEQRLSYETQ    SAAARVGMVL    SNADQLLSVV    VADESVLRLD    RDECTRFVGR
tr_K60__OYQ0909   LEQRLSYETQ    SAAARVGMVL    SNADQLLSVV    VADESVLRLD    RDECTRFVGR
tr_R_syzygii_R2   LEQRLSYETQ    SAATRVGMVL    SNADQLLSVV    VADESVLRLD    RDECTRFVSR
tr_Po82__tr_F6G   LEQRLSYETQ    SAAARVGMVL    SNADQLLSVV    VADESVLRLD    RDECTRFVGR
tr_UW551__EAP72   LEQRLSYETQ    SAAARVGMVL    SNADQLLSVV    VADESVLRLD    RDECTRFVGR
tr_PSI07__CBJ34   LEQRLSYETQ    SAATRVGMVL    SNADQLLSVV    VADESVLRLD    RDECTRFVSR
Consistency       **********    ***8******    **********    ****9*****    ********6*

                  ......... 160 ......... 170 ......... 180 ......... 190 ......... 200
tr_GMI1000__tr_   VIHNQSDFAT    LGVADAHGKL    VCTPVPGAIG    LDVADREFFR    ELIATGRPSL
tr_OE1-1__AVV67   VIHNQSDFAT    LGVADAHGKL    VCTPVPGAIG    LDVADREFFR    ELIATGRPSL
tr_CMR15__tr_D8   VIHNQSDFAT    LGVADAHGKL    VCTPVPGAIG    LDVADREFFR    ELIATGRPSL
tr_FQY_4_tr_M4U   VIHNQSDFAT    LGVADAHGKL    VCTPVPGAIG    LDVADREFFR    ELIATGRPSL
tr_CFBP2957__tr   VIHNQSDFAT    LGVADARGKL    ICTPAPGAIG    LDISDRDFFR    ELVATGRPSL
tr_UYO31__tr_A0   VIHNQSDFAT    LGVADARGKL    ICTPAPGAIG    LDISDRDFFR    ELIATGRPSL
tr_IPO1609__CEJ   VIHNQSDFAT    LGVADARGKL    ICTPAPGAIG    LDISDRDFFR    ELIATGRPSL
tr_K60__OYQ0909   VIHNQSDFAT    LGVADARGKL    ICTPAPGAIG    LDISDRDFFR    ELIATGRPSL
tr_R_syzygii_R2   VIHNQSDFAT    LGVADARGKL    VCTPVPGAIG    LDIADRDFFR    ELIATGRPSL
tr_Po82__tr_F6G   VIHNQSDFAT    LGVADARGKL    ICTPAPGAIG    LDISDRDFFR    ELIATGRPSL
tr_UW551__EAP72   VIHNQSDFAT    LGVADARGKL    ICTPAPGAIG    LDISDRDFFR    ELIATGRPSL
tr_PSI07__CBJ34   VIHNQSDFAT    LGVADARGKL    VCTPVPGAIG    LDIADRDFFR    ELIATGRPSL
Consistency       **********    ******7***    9***7*****    **97**8***    **9*******

                  ......... 210 ......... 220 ......... 230 ......... 240 ......... 250
tr_GMI1000__tr_   SNFLTGRTSH    QPVIVATRAV    VGPDGGIRGV    AYAAIRQSAL    MVAAGSASSG
tr_OE1-1__AVV67   SNFLTGRTSH    QPVIVATRAV    VGPDGGIRGV    AYAAIRQSAL    MVAAGSASSG
tr_CMR15__tr_D8   SNFLTGRTSH    QPVMVATRAV    VGPDGGILGV    AYAAIRQSAL    MVAAGSAASG
tr_FQY_4_tr_M4U   SNFLTGRTSH    QPVIVATRAV    VGPDGGIRGV    AYAAIRQSAL    MVAAGSAASG
```

```
tr_CFBP2957__tr SNFLTGRTSH QPVMVATRAV VGPDGAIRGV AYAAIRQGAL MVAAGGGASG
tr_UYO31__tr_A0 SNFLTGRTSH QPVMVATRAV VGPDGAIRGV AYAAIRQGAL TVAAGGGASG
tr_IPO1609__CEJ SNFLTGRTSH QPVMVATRAV VGPDGAIRGV AYAAIRQGAL TVAAGGGASG
tr_K60__OYQ0909 SNFLTGRTSH QPVMVATRAV VGPDGAIRGV AYAAIRQGAL MVAAGGGASG
tr_R_syzygii_R2 SNFLTGRTSH QPVMVATRAV VGPDGAILGV AYAAIRQSAL TVAAGSTTAG
tr_Po82__tr_F6G SNFLTGRTSH QPVMVATRAV VGPDGAIRGV AYAAIRQGAL TVAAGGGASG
tr_UW551__EAP72 SNFLTGRTSH QPVMVATRAV VGPDGAIRGV AYAAIRQGAL TVAAGGGASG
tr_PSI07__CBJ34 SNFLTGRTSH QPVMVATRAV VGPDGAILGV AYAAIRQSAL MVAAGSTTAG
Consistency     ********** ***8****** *****7*6** *******6** 6****6578*

                ......... 260 ......... 270 ......... 280 ......... 290 ......... 300
tr_GMI1000__tr_ PVYLVDGAGT VLSGTHAALS GEGPGVTRGD LVSAAGPEPG ATVFETDADG
tr_OE-1-1__AVV67 PVYLVDGAGT VLSGTHAALS GEGPGVTRGD LVSAAGPEPG ATVFETDADG
tr_CMR15__tr_D8 PVYLVDSAGT VLSGTHAAPS GEGPGVTRGD IVSAAGPEPG ATVFETDADG
tr_FQY_4_tr_M4U PVYLVDSAGT VLSGTHAAPS GEGPGVTRGD LVSAAGPEPG ATVFETDADG
tr_CFBP2957__tr PVYLVDSAGT VLSGPHANPS EDVPGVAKGD LVSAAGAEPG ATVFKTDPDG
tr_UYO31__tr_A0 PVYLVDSAGT VLSGTHANPS EEVPGVAKGD LVSAAGAEPG ATLFKTDPDG
tr_IPO1609__CEJ PVYLVDSAGT VLSGTHANPS EEVPGVAKGD LVSAAGAEPG ATLFKTDPDG
tr_K60__OYQ0909 PVYLVDSAGT VLSGPHANPS EDVPGVAKGD LVSAAGAEPG ATVFKTDPDG
tr_R_syzygii_R2 PVYLVDSAGT VLSGAHATQP GDGPGVTRSD LVSAAGPEPG ATVFETDPDG
tr_Po82__tr_F6G PVYLVDSAGT VLSGTHANPS EEVPGVAKGD LVSAAGAEPG ATLFKTDPDG
tr_UW551__EAP72 PVYLVDSAGT VLSGTHANPS EEVPGVAKGD LVSAAGAEPG ATLFKTDPDG
tr_PSI07__CBJ34 PVYLVDSAGT VLSGAHATQP GDGPGVTRSD LVSAAGPEPG ATVFETDPDG
Consistency     ******8*** ****6**557 585***788* 9*****6*** **8*7**6**

                ......... 310 ......... 320 ......... 330 ......... 340 ......... 350
tr_GMI1000__tr_ TLRAYAAMAV PHATSGRLRL VRGIDATDMA RQQRDVTLAG GGAIVLMLVL
tr_OE-1-1__AVV67 TLRAYAAMAV PHATSGRLRL VRGIDATDMA RQQRDVTLAG GGAIVLMLVL
tr_CMR15__tr_D8 TLRAYAAMAV PHVTSGRLRL VRGIDATDMA RQQRDVTLAG GGAIVLMLVL
tr_FQY_4_tr_M4U TLRAYAAMAV PHATSGRLRL VRGIDATDMA RQQRDVTLAG GGAIVLMLVL
tr_CFBP2957__tr TLRAYAAMAV PHVTSGRLRL VRGIDATDMA RQQRDVALAG GGAIGLMLVL
tr_UYO31__tr_A0 TLRAYAAMAV PHVTSGRLRL VRGIDATDMA RQQRDVALAG GGAIGLMLVL
tr_IPO1609__CEJ TLRAYAAMAV PHVTSGRLRL VRGIDATDMA RQQRDVALAG GGAIGLMLVL
tr_K60__OYQ0909 TLRAYAAMAV PHVTSGRLRL VRGIDATDMA RQQRDVALAG GGAIGLMLVL
tr_R_syzygii_R2 TLRAYAAMAV PHATGGRLRL VRGIDATDMA RQQRDVTLAG GGAIVLMLVL
tr_Po82__tr_F6G TLRAYAAMAV PHVTSGRLRL VRGIDATDMA RQQRDVALAG GGAIGLMLVL
tr_UW551__EAP72 TLRAYAAMAV PHVTSGRLRL VRGIDATDMA RQQRDVALAG GGAIGLMLVL
tr_PSI07__CBJ34 TLRAYAAMAV PHATSGRLRL VRGIDATDMA RQQRDVTLAG GGAIVLMLVL
Consistency     ********** **7*9***** ********** ******7*** ****5*****

                ......... 360 ......... 370 ......... 380 ......... 390 ......... 400
tr_GMI1000__tr_ LLALIQMAMR RLVLPRVDAL VDAARHYAAG DFSARVAEQG GARDELSLLE
tr_OE-1-1__AVV67 LLALIQMAMR RLVLPRVDAL VDAARHYAAG DFSARVAEQG GARDELSLLE
tr_CMR15__tr_D8 LLALIQMAMR RLVLPRVDAL VNAARHYAAG DFSTRVAEQG GARDELSLLE
tr_FQY_4_tr_M4U LLALIQMAMR RLVLPRVDAL VDAARHYAAG DFSARVAEQG GARDELSLLE
tr_CFBP2957__tr LLALIQMAMR RLVLPRVDAL VDAARRYAAG DFSTRVAEQG GARDELSLLE
tr_UYO31__tr_A0 LLALIQMAMR RLVLPRVDAL VDAARRYAAG DFSTRVAEQG GARDELSLLE
tr_IPO1609__CEJ LLALIQMAMR RLVLPRVDAL VDAARRYAAG DFSTRVAEQG GARDELSLLE
tr_K60__OYQ0909 LLALIQMAMW RLVLPRVDAL VDAARRYAAG DFSTRVAEQG GARDELSLLE
tr_R_syzygii_R2 LLALIQMAMR RLVLPRVDAL VDAARHYAAG DFSARVAEQG GARDELSLLE
tr_Po82__tr_F6G LLALIQMAMR RLVLPRVDAL VDAARRYAAG DFSTRVAEQG GARDELSLLE
tr_UW551__EAP72 LLALIQMAMR RLVLPRVDAL VDAARRYAAG DFSTRVAEQG GARDELSLLE
tr_PSI07__CBJ34 LLALIQMAMR RLVLPRVDAL VDAARHYAAG DFSTRVAEQG GARDELSLLE
Consistency     *********8 ********** *9***6**** ***7****** **********

                ......... 410 ......... 420 ......... 430 ......... 440 ......... 450
tr_GMI1000__tr_ RTFNEMRTAI LRHDETVHHL TERFQRVARA TNDWIFDWDI ATGESWANAS
tr_OE-1-1__AVV67 RTFNEMRTAI LRHDETVHHL TERFQRVARA TNDWIFDWDI ATGESWANAS
tr_CMR15__tr_D8 RTFNEMRTAI LRHDETVHHL TERFQRVARA TNDWIFDWDI ATGESWANAS
tr_FQY_4_tr_M4U RTFNEMRTAI LRHDETVHHL TERFQRVARA TNDWIFDWDI ATGESWANAS
tr_CFBP2957__tr RTFNEMRMAI QRHDETVHHL TERFQRVARA TNDWIFDWDI ATGESWANAS
tr_UYO31__tr_A0 RTFNEMRMAI QRHDETVHHL TERFQRVARA TNDWIFDWDI ATGESWANAS
tr_IPO1609__CEJ RTFNEMRMAI QRHDETVHHL TERFQRVARA TNDWIFDWDI ATGESWANAS
tr_K60__OYQ0909 RTFNEMRMAI QRHDETVHHL TERFQRVARA TNDWIFDWDI ATGESWANAS
tr_R_syzygii_R2 RTFNEMRMAI LRHDETVHHL TERFQRVARA TNDWIFDWDI ATGESWVNAS
tr_Po82__tr_F6G RTFNEMRMAI QRHDETVHHL TERFQRVARA TNDWIFDWDI ATGESWANAS
tr_UW551__EAP72 RTFNEMRMAI QRHDETVHHL TERFQRVARA TNDWIFDWDI ATGESWANAS
tr_PSI07__CBJ34 RTFNEMRMAI LRHDETVHHL TERFQRVARA TNDWIFDWDI ATGESWANAS
Consistency     *******6** 5********* ********** ********** ******9***

                ......... 460 ......... 470 ......... 480 ......... 490 ......... 500
tr_GMI1000__tr_ LHRLLGSDAL LAQGDEDGVS RTLTFQQFVH PEDMAAFGRG LRAALHSDRD
tr_OE-1-1__AVV67 LHRLLGSDAL LAQGDEDGVS RTLTFQQFVH PEDMAAFGRG LRAALHSDRD
tr_CMR15__tr_D8 LHRLLGSDAL LAQGDEDGVS RTLTFQQFVH PEDMEAFGRG LRAALHSDRD
tr_FQY_4_tr_M4U LHRLLGSDAL LAQGDEDGVS RTLTFQQFVH PEDMAAFGRG LRAALHSDRD
```

```
tr_CFBP2957__tr LHRLLGSDAL LTPGDEDGVS RTLTFQQFVH PEDMEVFGRG LRAALHSDRT
tr_UY031__tr_A0 LHRLLGSDAL LTSGDEDGVS RTLTFQQFVH PEDMEVFGRG LRAALHSDRN
tr_IPO1609__CEJ LHRLLGSDAL LTSGDEDGVS RTLTFQQFVH PEDMEVFGRG LRAALHSDRN
tr_K60__OYQ0909 LHRLLGSDAQ LTPGDEDGVS RTLTFQQFVH PEDMEVFGRG LRAALHSDRN
tr_R_syzygii_R2 LHRLLGSDAL LASGDEDGVS RTLTFQQFVH PEDMEAFGWG LRAALHSDRN
tr_Po82__tr_F6G LHRLLGSDAL LTSGDEDGVS RTLTFQQFVH PEDMEVFGRG LRAALHSDRN
tr_UW551__EAP72 LHRLLGSDAL LTSGDEDGVS RTLTFQQFVH PEDMEVFGRG LRAALHSDRN
tr_PSI07__CBJ34 LHRLLGSDAL LASGDEDGVS RTLTFQQFVH PEDMEAFGWG LRAALHSDRN
Consistency     *********8 *75******* ********** ****77**7* *********6

                .........510 .........520 .........530 .........540 .........550
tr_GMI1000__tr_ AWHHVCRVID ASRAVRTVEI RASIYRGKDG RAVRMVGGVT DISQRSAMEA
tr_OE1-1__AVV67 AWHHVCRVID ASRAVRTVEI RASIYRGKDG RAVRMVGGVT DISQRSAMEA
tr_CMR15__tr_D8 AWHHVCRVID ASRAVRTVEI RASIYRGKDG RALRMVGGVT DISQRSAMEA
tr_FQY_4_tr_M4U AWHHVCRVID ASRAVRTVEI RASIYRGKDG RAVRMVGGVT DISQRSAMEA
tr_CFBP2957__tr AWHHVCRVID ASRAVRTVEI RASIYRGKDG RAMRMVGGVT DISQRSAMEA
tr_UY031__tr_A0 AWHHVCRVID ASRAVRTVEI RASIYRGKDG RAMRMVGGVT DISQRSAMEA
tr_IPO1609__CEJ AWHHVCRVID ASRAVRTVEI RASIYRGKDG RAMRMVGGVT DISQRSAMEA
tr_K60__OYQ0909 AWHHVCRVID ASRAVRTVEI RASIYRGKDG RAMRMVGGVT DISQRSAMEA
tr_R_syzygii_R2 AWHHVCRVID ASRAVRTVEI RASIYRGEDG RAMRMVGGVT DISQRSAMEA
tr_Po82__tr_F6G AWHHVCRVID ASRAVRTVEI RASIYRGKDG RAMRMVGGVT DISQRSAMEA
tr_UW551__EAP72 AWHHVCRVID ASRAVRTVEI RASIYRGKDG RAMRMVGGVT DISQRSAMEA
tr_PSI07__CBJ34 AWHHVCRVID ASRAVRTVEI RASIYRGKDG RAMRMVGGVT DISQRSAMEA
Consistency     ********** ********** *******9** **7******* **********

                .........560 .........570 .........580 .........590 .........600
tr_GMI1000__tr_ DLRASEANLR VAEQIALLGS WRWDVLRDTA TWSSGMYLLT GVSPGPPPSF
tr_OE1-1__AVV67 DLRASEANLR VAEQIALLGS WRWDVLRDTA TWSSGMYLLT GVSPGPPPSF
tr_CMR15__tr_D8 DLRASEANLR VAEQIALLGS WRWDVLRDTA TWSSGMYLLT GVPPGPPPSF
tr_FQY_4_tr_M4U DLRASEANLR VAEQIALLGS WRWDVLRDTA TWSSGMYLLT GVSPGPPPSF
tr_CFBP2957__tr ELRASEANLR VAEQIALLGS WRWDVLRDTA TWSTGMYVLT GMPPGPPPSF
tr_UY031__tr_A0 DLRASEANLR VAEQIALLGS WRWDVLRDTA TWSTGMYVLT CMPPGPPPSF
tr_IPO1609__CEJ DLRASEANLR VAEQIALLGS WRWDVLRDTA TWSTGMYVLT CMPPGPPPSF
tr_K60__OYQ0909 DLRASEANLR VAEQIALLGS WRWDVLRDTA TWSTGMYVLT GVSPGPPPSF
tr_R_syzygii_R2 DLRASEANLR VAEQIALLGS WRWDVLRDTA TWSSGMYVLT GVPPGPPPSF
tr_Po82__tr_F6G DLRASEANLR VAEQIALLGS WRWDVLRDTA TWSTGMYVLT GMPPGPPPSF
tr_UW551__EAP72 DLRASEANLR VAEQIALLGS WRWDVLRDTA TWSTGMYVLT CMPPGPPPSF
tr_PSI07__CBJ34 DLRASEANLR VAEQIALLGS WRWDVLRDTA TWSSGMYVLT GVPPGPPPSF
Consistency     9********* ********** ********** ***7***8** 676*******

                .........610 .........620 .........630 .........640 .........650
tr_GMI1000__tr_ AQQAQFFTGD SYNRLREAAS RAVTEGVPYS LELEMIRRDG EHRWVLSRGN
tr_OE1-1__AVV67 AQQAQFFTGD SYNRLREAAS RAVTEGVPYS LELEMIRRDG EHRWVLSRGN
tr_CMR15__tr_D8 AQQAQFFTGD SYNRLREAAS RAVTEGVPYS LELEMIRRDG EHRWVLSRGN
tr_FQY_4_tr_M4U AQQAQFFTGD SYNRLREAAS RAVTEGVPYS LELEMIRRDG EHRWVLSRGN
tr_CFBP2957__tr AQQAQFFTAD SYNRLREAAS RAVTEGEPYS LELEMIRRDG EHRWVLSRGN
tr_UY031__tr_A0 AQQAQFFTAD SYNRLREAAS RAVTEGEPYS LELEMIRRDG EHRWVLSRGN
tr_IPO1609__CEJ AQQAQFFTAD SYNRLREAAS RAVTEGEPYS LELEMIRRDG EHRWVLSRGN
tr_K60__OYQ0909 EQQAQFFTAD SYNRLREAAS RAVTEGEPYS LELEMIRRDG EHRWVLSRGN
tr_R_syzygii_R2 AQQAQFFTGD SYNRLREAAS RAVTEGEPYS LELEMIRRDG EHRWVLSRGN
tr_Po82__tr_F6G AQQAQFFTAD SYNRLREAAS RAVTEGEPYS LELEMIRRDG EHRWVLSRGN
tr_UW551__EAP72 AQQAQFFTAD SYNRLREAAS RAVTEGEPYS LELEMIRRDG EHRWVLSRGN
tr_PSI07__CBJ34 AQQAQFFTGD SYNRLREAAS RAVTEGEPYS LELEMIRRDG EHRWVLSRGN
Consistency     8*******6* ********** ******6*** ********** **********

                .........660 .........670 .........680 .........690 .........700
tr_GMI1000__tr_ IERNEREEVV ALFGTMQDIT ERRESDEQLR LLRRVVESVP SGITVADAQQ
tr_OE1-1__AVV67 IERNEREEVV ALFGTMQDIT ERRESDEQLR LLRRVVESVP SGITVADAQQ
tr_CMR15__tr_D8 IERNEREEVV ALFGTMQDIT ERRESDEQLR LLRRVVESVP SGITVADAQQ
tr_FQY_4_tr_M4U IERNEREEVV ALFGTMQDIT ERRESDEQLR LLRRVVESVP SGITVADAQQ
tr_CFBP2957__tr IERNGREEVV ALFGTMQDIT ERRESDEQLR LLRRVVESVP SGISVADALQ
tr_UY031__tr_A0 IERNEREEVV ALFGTMQDIT ERRESDEQLR LLRRVVESVP SGISVADALQ
tr_IPO1609__CEJ IERNEREEVV ALFGTMQDIT ERRESDEQLR LLRRVVESVP SGISVADALQ
tr_K60__OYQ0909 IERNEREEVV ALFGTMQDIT ERRESDEQLR LLRRVVESVP SGITVADALQ
tr_R_syzygii_R2 IERNEREEVV ALFGTMQDIT ERRESDEQLR LLRRVVESVP SGITVADALQ
tr_Po82__tr_F6G IERNEREEVV ALFGTMQDIT ERRESDEQLR LLRRVVESVP SGISVADALQ
tr_UW551__EAP72 IERNEREEVV ALFGTMQDIT ERRESDEQLR LLRRVVESVP SGISVADALQ
tr_PSI07__CBJ34 IERNEREEVV ALFGTMQDIT ERRESDEQLR LLRRVVESVP SGITVADALQ
Consistency     ****8***** ********** ********** ********** ***7****6*

                .........710 .........720 .........730 .........740 .........750
tr_GMI1000__tr_ PDLPLVYVNP GFERMTGYRA EEVLGRNCRF LHSSEPGQPA LNEVRAALRD
tr_OE1-1__AVV67 PDLPLVYVNP GFERMTGYRA EEVLGRNCRF LHSSEPGQPA LNEVRAALRD
tr_CMR15__tr_D8 PDLPLVYVNP GFERMTGYRA EEVLGRNCRF LHSSEPGQPA LNEVRAALRD
tr_FQY_4_tr_M4U PDLPLVYVNP GFERMTGYRA EEVLGRNCRF LHSSEPGQPA LNEVRAALRD
```

```
tr_CFBP2957__tr PDLPLVYVNP GFERMTGYRA EEVLGRNCRF LHSSEPGQPA LNEVRTALRD
tr_UYO31__tr_A0 PDLPLVYVNP GFERMTGYRA EEVLGRNCRF LHSSEPGQPA LNEVRTALRD
tr_IPO1609__CEJ PDLPLVYVNP GFERMTGYRA EEVLGRNCRF LHSSEPGQPA LNEVRTALRD
tr_K60__OYQ0909 PDLPLVYVNP GFERMTGYRA EEVLGRNCRF LHSSEPGQPA LNEVRTALRD
tr_R_syzygii_R2 PDLPLVYVNP GFERMTGYRA EEVLGRNCRF LHSSEPGQPA LNEVRAALRD
tr_Po82__tr_F6G PDLPLVYVNP GFERMTGYRA EEVLGRNCRF LHSSEPGQPA LNEVRTALRD
tr_UW551__EAP72 PDLPLVYVNP GFERMTGYRA EEVLGRNCRF LHSSEPGQPA LNEVRTALRD
tr_PSI07__CBJ34 PDLPLVYVNP GFERMTGYRA EEVLGRNCRF LHSSEPGQPA LNEVRAALRD
Consistency     ********** ********** ********** ********** *****7****

                . . . . . . . . . 760 . . . . . . . . . 770 . . . . . . . . . 780 . . . . . . . . . 790 . . . . . . . . . 800
tr_GMI1000__tr_ ASEIRVLLRN FRKDGHAFLN NFLLSPVRDS QGAVTHYVGI QDDVTEQEMT
tr_OE1-1__AVV67 ASEIRVLLRN FRKDGHAFLN NFLLSPVRDS QGAVTHYVGI QDDVTEQEMT
tr_CMR15__tr_D8 ASEIRVLLRN FRKDGHAFLN NFLLSPVRDS QGAVTHYVGI QDDVTEQEMT
tr_FQY_4_tr_M4U ASEIRVLLRN FRKDGHAFLN NFLLSPVRDS QGVVTHYVGI QDDVTEQEMT
tr_CFBP2957__tr ASEIRVLLRN FRKDGHPFLN NFLLSPVRDS RGAVTHYVGI QDDVTEQEMT
tr_UYO31__tr_A0 ASEIRVLLRN FRKDGHPFLN NFLLSPVRDS RGAVTHYVGI QDDVTEQEMT
tr_IPO1609__CEJ ASEIRVLLRN FRKDGHPFLN NFLLSPVRDS RGAVTHYVGI QDDVTEQEMT
tr_K60__OYQ0909 ASEIRVLLRN FRKDGHPFLN NFLLSPVRDS RGAVTHYVGI QDDVTEQEMT
tr_R_syzygii_R2 ASEIRVLLRN FRKDGHSFLN NFLLSPVRDS QGAVTHYVGI QDDVTEQEMT
tr_Po82__tr_F6G ASEIRVLLRN FRKDGHPFLN NFLLSPVRDS RGAVTHYVGI QDDVTEQEMT
tr_UW551__EAP72 ASEIRVLLRN FRKDGHPFLN NFLLSPVRDS RGAVTHYVGI QDDVTEQEMT
tr_PSI07__CBJ34 ASEIRVLLRN FRKDGHSFLN NFLLSPVRDS QGAVTHYVGI QDDVTEQEMT
Consistency     ********** ******5*** ********** 7*9******* **********

                . . . . . . . . . 810 . . . . . . . . . 820 . . . . . . . . . 830 . . . . . . . . . 840 . . . . . . . . . 850
tr_GMI1000__tr_ RARLAQHATV DPLTGLPNRT LLADRVQQGV EMAARQRSRF YVALINIDRF
tr_OE1-1__AVV67 RARLAQHATV DPLTGLPNRT LLADRVQQGV EMAARQRSRF YVALINIDRF
tr_CMR15__tr_D8 RARLAQHATV DPLTGLPNRT LLADRVQQGV EMAARQRSRF YVALINIDRF
tr_FQY_4_tr_M4U RARLAQHATV DPLTGLPNRT LLADRVQQGV EMAARQRSRF YVALINIDRF
tr_CFBP2957__tr RARLAQHATV DPLTGLPNRT LLADRVQQAV EMAARQRSRF YVALINIDRF
tr_UYO31__tr_A0 RARLAQHATV DPLTGLPNRT LLADRVQQAV EMAARQRSRF YVALINIDRF
tr_IPO1609__CEJ RARLAQHATV DPLTGLPNRT LLADRVQQAV EMAARQRSRF YVALINIDRF
tr_K60__OYQ0909 RARLAQHATV DPLTGLPNRT LLADRVQQAV EMAARQRSRF YVALINIDRF
tr_R_syzygii_R2 RARLAQHATA DPLTGLPNRT LLADRVQQAV EMAARQRSRF YVALINIDRF
tr_Po82__tr_F6G RARLAQHATV DPLTGLPNRT LLADRVQQAV EMAARQRSRF YVALINIDRF
tr_UW551__EAP72 RARLAQHATV DPLTGLPNRT LLADRVQQAV EMAARQRSRF YVALINIDRF
tr_PSI07__CBJ34 RARLAQHATA DPLTGLPNRT LLADRVQQAV EMAARQRSRF YVALINIDRF
Consistency     *********8 ********** ********7* ********** **********

                . . . . . . . . . 860 . . . . . . . . . 870 . . . . . . . . . 880 . . . . . . . . . 890 . . . . . . . . . 900
tr_GMI1000__tr_ KVVNDSLGHL LGDEVLRRIA ERLRDAADTV DTVARFGGDV FALVISHAGS
tr_OE1-1__AVV67 KVVNDSLGHL LGDEVLRRIA ERLRDAADTV DTVARFGGDV FALVISHAGS
tr_CMR15__tr_D8 KVVNDSLGHL LGDEVLRRIA ERLRDAADTV DTVARFGGDV FALVISHAGS
tr_FQY_4_tr_M4U KVVNDSLGHL LGDEVLRRIA ERLRDAADTV DTVARFGGDV FALVISHAGS
tr_CFBP2957__tr KVVNDSLGHL LGDEVLRRIA ERLRDAADTV DTVARFGGDV FALVISHAGS
tr_UYO31__tr_A0 KVVNDSLGHL LGDEVLRRIA ERLRDAADTV DTVARFGGDV FALVISHAGS
tr_IPO1609__CEJ KVVNDSLGHL LGDEVLRRIA ERLRDAADTV DTVARFGGDV FALVISHAGS
tr_K60__OYQ0909 KVVNDSLGHL LGDEVLRRIA ERLRDAADTV DTVARFGGDV FALVISHAGS
tr_R_syzygii_R2 KVVNDSLGHL LGDEVLRRVA ERLRDAADTV DTVARFGGDV FALVISHAGS
tr_Po82__tr_F6G KVVNDSLGHL LGDEVLRRIA ERLRDAADTV DTVARFGGDV FALVISHAGS
tr_UW551__EAP72 KVVNDSLGHL LGDEVLRRIA ERLRDAADTV DTVARFGGDV FALVISHAGS
tr_PSI07__CBJ34 KVVNDSLGHL LGDEVLRRVA ERLRDAADTV DTVARFGGDV FALVISHAGS
Consistency     ********** ********9* ********** ********** **********

                . . . . . . . . . 910 . . . . . . . . . 920 . . . . . . . . . 930 . . . . . . . . . 940 . . . . . . . . . 950
tr_GMI1000__tr_ HGTDLGFDLF AEPIRVEGHE VFVTASIGVA EYPAHGSDSE TLVRHAEMAM
tr_OE1-1__AVV67 HGTDLGFDLF AEPIRVEGHE VFVTASIGVA EYPAHGSDSE TLVRHAEMAM
tr_CMR15__tr_D8 HGTDLGFDLF AEPIRVEGHE VFVTASIGVA EYPAHGSDSE TLVRHAEMAM
tr_FQY_4_tr_M4U HGTDLGFDLF AEPIRVEGHE VFVTASIGVA EYPAHGSDSE TLVRHAEMAM
tr_CFBP2957__tr HGVDLGFDLF AEPIRVEGHE VFVTASIGVA EYPAHGTDSE TLVRHAEMAM
tr_UYO31__tr_A0 HGVDLGFDLF AEPIRVEGHE VFVTASIGVA EYPAHGTDSE TLVRHAEMAM
tr_IPO1609__CEJ HGVDLGFDLF AEPIRVEGHE VFVTASIGVA EYPAHGTDSE TLVRHAEMAM
tr_K60__OYQ0909 HGVDLGFDLF AEPIRVEGHE VFVTASIGVA EYPAHGTDSE TLVRHAEMAM
tr_R_syzygii_R2 HGVDLGFDLF AEPIRVEGHE VFVTASIGVA EYPAHGSNSE TLVRHAEMAM
tr_Po82__tr_F6G HGVDLGFDLF AEPIRVEGHE VFVTASIGVA EYPAHGADSE TLVRHAEMAM
tr_UW551__EAP72 HGVDLGFDLF AEPIRVEGHE VFVTASIGVA EYPAHGTDSE TLVRHAEMAM
tr_PSI07__CBJ34 HGVDLGFDLF AEPIRVEGHE VFVTASIGVA EYPAHGSNSE TLVRHAEMAM
Consistency     **7******* ********** ********** ******78** **********

                . . . . . . . . . 960 . . . . . . . . . 970 . . . . . . . . . 980 . . . . . . . . . 990 . . . . . . . . . 1000
tr_GMI1000__tr_ YFAKQNGRNR LEFFAPEMDI GVSYRLNLEH QIRAALEQGQ FRLLYQPQID
tr_OE1-1__AVV67 YFAKQNGRNR LEFFAPEMDI GVSYRLNLEH QIRAALEQGQ FRLLYQPQID
tr_CMR15__tr_D8 YFAKQNGRNR LEFFAPEMDI GVSYRLNLEH QIRAALEQGQ FRLLYQPQID
tr_FQY_4_tr_M4U YFAKQNGRNR LEFFAPEMDI GVSYRLNLEH QIRAALEQGQ FRLLYQPQID
```

```
tr_CFBP2957__tr YYAKQNGRNR LEFFAPEMDI GVSYRLNLEH QIRAALEHGQ FRLLYQPQID
tr_UY031__tr_A0 YYAKQNGRNR LEFFAPEMDI GVSYRLNLEH QIRAALEHGQ FRLLYQPQID
tr_IPO1609__CEJ YYAKQNGRNR LEFFAPEMDI GVSYRLNLEH QIRAALEHGQ FRLLYQPQID
tr_K60__OYQ0909 YYAKQNGRNR LEFFAPEMDI GVSYRLNLEH QIRAALEHGQ FRLLYQPQID
tr_R_syzygii_R2 YYAKQNGRNR LEFFAPEMDI GVSYRLNLEH QIRAALEQGQ FRLLYQPQID
tr_Po82__tr_F6G YYAKQNGRNR LEFFAPEMDI GVSYRLNLEH QIRAALEHGQ FRLLYQPQID
tr_UW551__EAP72 YYAKQNGRNR LEFFAPEMDI GVSYRLNLEH QIRAALEHGQ FRLLYQPQID
tr_PSI07__CBJ34 YYAKQNGRNR LEFFAPEMDI GVSYRLNLEH QIRAALEQGQ FRLLYQPQID
Consistency     *8******** ********** ********** *******6** **********

                .........1010.........1020.........1030.........1040.........1050
tr_GMI1000__tr_ TKTGRICGLE ALIRWIHPTR GLLPPAAFIP VAEESGLVVE IGNWVLAEAA
tr_OE1-1__AVV67 TKTGRICGLE ALIRWIHPTR GLLPPAAFIP VAEESGLVVE IGNWVLAEAA
tr_CMR15__tr_D8 TKTGRICGLE ALIRWIHPTR GLLPPAAFIP VAEESGLVVE IGNWVLAEAA
tr_FQY_4_tr_M4U TKTGRICGLE ALIRWIHPTR GLLPPAAFIP VAEESGLVVE IGNWVLAEAA
tr_CFBP2957__tr TRTGRICGLE ALIRWIHPTR GQLPPAAFIP VAEESGLVVE IGNWVLSEAA
tr_UY031__tr_A0 TRTGRICGLE ALIRWIHPTR GQLPPAAFIP VAEESGLVVE IGNWVLSEAA
tr_IPO1609__CEJ TRTGRICGLE ALIRWIHPTR GQLPPAAFIP VAEESGLVVE IGNWVLSEAA
tr_K60__OYQ0909 TRTGRICGLE ALIRWIHPTR GQLPPAAFIP VAEESGLVVE IGNWVLSEAA
tr_R_syzygii_R2 SRTGRICGLE ALIRWIHPTR GQLPPAAFIP VAEESGLVVE IGNWVLAEAA
tr_Po82__tr_F6G TRTGRICGLE ALIRWIHPTR GQLPPAAFIP VAEESGLVVE IGNWVLSEAA
tr_UW551__EAP72 TRTGRICGLE ALIRWIHPTR GQLPPAAFIP VAEESGLVVE IGNWVLSEAA
tr_PSI07__CBJ34 SRTGRICGLE ALIRWIHPTR GQLPPAAFIP VAEESGLVVE IGNWVLAEAA
Consistency     88******** ********** *6******** ********** ******7***

                .........1060.........1070.........1080.........1090.........1100
tr_GMI1000__tr_ QQRAAWHARG LGEDLTIAVN VSPLQFKRGT VLPTLLRLQR QHGLGSGFLE
tr_OE1-1__AVV67 QQRAAWHARG LGEDLTIAVN VSPLQFKRGT VLPTLLRLQR QHGLGSGFLE
tr_CMR15__tr_D8 QQRAAWHARG LGEDLTIAVN VSPLQFKRGT VLPTLLRLQR QHGLGSGFLE
tr_FQY_4_tr_M4U QQRAAWHARG LGEDLTIAVN VSPLQFKRGT VLPTLLRLQR QHGLGSGFLE
tr_CFBP2957__tr QQRAAWHARG LGEDLTIAVN VSPLQFKRGT VLPTLLRLQR QHGLGSGFLE
tr_UY031__tr_A0 QQRAAWHARG LGEDLTIAVN VSPLQFKRGT VLPTLLRLQR QHGLGSGFLE
tr_IPO1609__CEJ QQRAAWHARG LGEDLTIAVN VSPLQFKRGT VLPTLLRLQR QHGLGSGFLE
tr_K60__OYQ0909 QQRAAWHARG LGEDLTIAVN VSPLQFKRGT VLPTLLRLQR QHGLGSGFLE
tr_R_syzygii_R2 QQRAAWHARG LGEDLTIAVN VSPLQFKRGT VLPTLLRLQR QHGLGSGFLE
tr_Po82__tr_F6G QQRAAWHARG LGEDLTIAVN VSPLQFKRGT VLPTLLRLQR QHGLGSGFLE
tr_UW551__EAP72 QQRAAWHARG LGEDLTIAVN VSPLQFKRGT VLPTLLRLQR QHGLGSGFLE
tr_PSI07__CBJ34 QQRAAWHARG LGEDLTIAVN VSPLQFKRGT VLPTLLRLQR QHGLGSGFLE
Consistency     ********** ********** ********** ********** **********

                .........1110.........1120.........1130.........1140.........1150
tr_GMI1000__tr_ LEVTESMLME GTERTIEDLT AIRQLGVRIA IDDFGTGYSS LAYLKRLPID
tr_OE1-1__AVV67 LEVTESMLME GTERTIEDLT AIRQLGVRIA IDDFGTGYSS LAYLKRLPID
tr_CMR15__tr_D8 LEVTESMLME GTERTIEDLT AIRQLGVRIA IDDFGTGYSS LAYLKRLPID
tr_FQY_4_tr_M4U LEVTESMLME GTERTIEDLT AIRQLGVRIA IDDFGTGYSS LAYLKRLPID
tr_CFBP2957__tr LEVTESMLME GTERTIEDLT AIRQLGVRIA IDDFGTGYSS LAYLKRLPID
tr_UY031__tr_A0 LEVTESMLME GTERTIEDLT AIRQLGVRIA IDDFGTGYSS LAYLKRLPID
tr_IPO1609__CEJ LEVTESMLME GTERTIEDLT AIRQLGVRIA IDDFGTGYSS LAYLKRLPID
tr_K60__OYQ0909 LEVTESMLME GTERTIEDLT AIRQLGVRIA IDDFGTGYSS LAYLKRLPID
tr_R_syzygii_R2 LEVTESMLME GTERTIEDLT AIRQLGVRIA IDDFGTGYSS LAYLKRLPID
tr_Po82__tr_F6G LEVTESMLME GTERTIEDLT AIRQLGVRIA IDDFGTGYSS LAYLKRLPID
tr_UW551__EAP72 LEVTESMLME GTERTIEDLT AIRQLGVRIA IDDFGTGYSS LAYLKRLPID
tr_PSI07__CBJ34 LEVTESMLME GTERTIEDLT AIRQLGVRIA IDDFGTGYSS LAYLKRLPID
Consistency     ********** ********** ********** ********** **********

                .........1160.........1170.........1180.........1190.........1200
tr_GMI1000__tr_ LIKIDRAFVK DIDRDSNDAA ICTTVVVLAH NLGVKVCAEG VEDAAQSAFL
tr_OE1-1__AVV67 LIKIDRAFVK DIDRDSNDAA ICTTVVVLAH NLGVKVCAEG VEDAAQSAFL
tr_CMR15__tr_D8 LIKIDRAFVK DIDRDSNDAA ICTTVVVLAH NLGVKVCAEG VEDAAQSAFL
tr_FQY_4_tr_M4U LIKIDRAFVK DIDRDSNDAA ICTTVVVLAH NLGVKVCAEG VEDAAQSAFL
tr_CFBP2957__tr LIKIDRAFVK DIDRDSNDAA ICTTVVVLAH NLGVKVCAEG VEDAAQSAFL
tr_UY031__tr_A0 LIKIDRAFVK DIDRDSNDAA ICTTVVVLAH NLGVKVCAEG VEDAAQSAFL
tr_IPO1609__CEJ LIKIDRAFVK DIDRDSNDAA ICTTVVVLAH NLGVKVCAEG VEDAAQSAFL
tr_K60__OYQ0909 LIKIDRAFVK DIDRDSNDAA ICTTVVVLAH NLGVKVCAEG VEDAAQSAFL
tr_R_syzygii_R2 LIKIDRAFVK DIDRDSNDAA ICTTVVVLAH NLGVKVCAEG VEDAAQSAFL
tr_Po82__tr_F6G LIKIDRAFVK DIDRDSNDAA ICTTVVVLAH NLGVKVCAEG VEDAAQSAFL
tr_UW551__EAP72 LIKIDRAFVK DIDRDSNDAA ICTTVVVLAH NLGVKVCAEG VEDAAQSAFL
tr_PSI07__CBJ34 LIKIDRAFVK DIDRDSNDAA ICTTVV---- ---------- ----------
Consistency     ********** ********** ******8888 8888888888 8888888888

                .........1210.........1220.........1230....
tr_GMI1000__tr_ ASHQCDVLQG YYFSEPLLPE AVTALLERDA RFTV
tr_OE1-1__AVV67 ASHQCDVLQG YYFSEPLLPE AVTALLERDA RFTV
tr_CMR15__tr_D8 ASHQCDVLQG YYFSEPLLPE AVTALLERDA RFTV
tr_FQY_4_tr_M4U ASHQCDVLQG YYFSEPLLPE AVTALLERDA RFTV
```

tr_CFBP2957__tr	ASHQCDVLQG	YYFSEPLLPD	AVTALLERDA	RFTV
tr_UYO31__tr_A0	ASHQCDVLQG	YYFSEPLLPD	AVTALLERDA	RFTV
tr_IPO1609__CEJ	ASHQCDVLQG	YYFSEPLLPD	AVTALLERDA	RFTV
tr_K60__OYQ0909	ASHQCDVLQG	YYFSEPLLPD	AVTALLERDA	RFTV
tr_R_syzygii_R2	ASHQCDVLQG	YYFSEPLLPD	AVTALLERDA	RFTV
tr_Po82__tr_F6G	ASHQCDVLQG	YYFSEPLLPD	AVTALLERDA	RFTV
tr_UW551__EAP72	ASHQCDVLQG	YYFSEPLLPD	AVTALLERDA	RFTV
tr_PSI07__CBJ34	----------	----------	----------	----
Consistency	8888888888	8888888886	8888888888	8888
