# Supplementary material for: Light modulates important physiological features of Ralstonia pseudosolanacearum during the colonization of tomato plants
Source: Sci Rep. 2021 Jul 15;11:14531. doi: 10.1038/s41598-021-93871-9 (PMC8282871; doi:10.1038/s41598-021-93871-9)
Supplement: Supplementary file 3 — Supplementary Information 3. [file 41598_2021_93871_MOESM3_ESM.docx]

**Light modulates important physiological features of *Ralstonia pseudosolanacearum* during the colonization of tomato plants.**

Josefina Tano^1ǁ^, María Belén Ripa^1ǁ^, María Laura Tondo^2^, Analía Carrau^1^, Silvana Petrocelli^2^, María Victoria Rodriguez^3^, Virginia Ferreira^4^, María Inés Siri^4^, Laura Piskulic^5^, Elena Graciela Orellano^1*^.

^1^Instituto de Biología Molecular y Celular de Rosario, Facultad de Ciencias Bioquímicas y Farmacéuticas, Universidad Nacional de Rosario, (IBR-CONICET-UNR), Rosario, Argentina.

^2^Facultad de Ciencias Bioquímicas y Farmacéuticas, Universidad Nacional de Rosario, Rosario, Argentina.

^3^Área Biología Vegetal (CONICET), Facultad de Ciencias Bioquímicas y Farmacéuticas, Universidad Nacional de Rosario, Rosario, Argentina.

^4^Área Microbiología, Departamento de Biociencias, Facultad de Química, Universidad de la República, Montevideo, Uruguay.

^5^Área estadística y procesamiento de datos, Facultad de Ciencias Bioquímicas y Farmacéuticas, Universidad Nacional de Rosario, Rosario, Argentina.

^ǁ^ These authors contributed equally to the work.

*To whom correspondence should be addressed at: Elena G. Orellano, IBR-CONICET-UNR, FCByF-UNR, Suipacha 531, (S2002LRK) Rosario, Argentina. Phone: +54 341 4350661. FAX: +54 341 4390465. Email: orellano@ibr-conicet.gov.ar


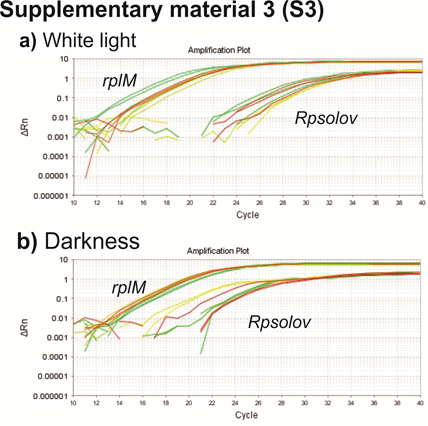


**Supplementary material S3. Amplification plot of gene expression data by quantitative real-time RT-PCR (RT-qPCR).** Amplification plot of gene expression data by quantitative real-time RT-PCR. The expression of the *Rpsolov* gene was assayed by RT-qPCR in the *Rpso* GMI1000 strain under different lighting conditions: **a)** White light and **b)** Darkness. Experimental raw data are shown in the software StepOne of the *Rpsolov* gene and the *rplM* reference gene in both lighting conditions tested.
